# Systematic review of the health benefits of physical activity and fitness in school-aged children and youth

**DOI:** 10.1186/1479-5868-7-40

**Published:** 2010-05-11

**Authors:** Ian Janssen, Allana G LeBlanc

**Affiliations:** 1 School of Kinesiology and Health Studies, Queen's University, Kingston, Ontario, Canada; 2 Department of Community Health and Epidemiology, Queen's University, Kingston, Ontario, Canada

## Abstract

**Background:**

The purpose was to: 1) perform a systematic review of studies examining the relation between physical activity, fitness, and health in school-aged children and youth, and 2) make recommendations based on the findings.

**Methods:**

The systematic review was limited to 7 health indicators: high blood cholesterol, high blood pressure, the metabolic syndrome, obesity, low bone density, depression, and injuries. Literature searches were conducted using predefined keywords in 6 key databases. A total of 11,088 potential papers were identified. The abstracts and full-text articles of potentially relevant papers were screened to determine eligibility. Data was abstracted for 113 outcomes from the 86 eligible papers. The evidence was graded for each health outcome using established criteria based on the quantity and quality of studies and strength of effect. The volume, intensity, and type of physical activity were considered.

**Results:**

Physical activity was associated with numerous health benefits. The dose-response relations observed in observational studies indicate that the more physical activity, the greater the health benefit. Results from experimental studies indicate that even modest amounts of physical activity can have health benefits in high-risk youngsters (e.g., obese). To achieve substantive health benefits, the physical activity should be of at least a moderate intensity. Vigorous intensity activities may provide even greater benefit. Aerobic-based activities had the greatest health benefit, other than for bone health, in which case high-impact weight bearing activities were required.

**Conclusion:**

The following recommendations were made: 1) Children and youth 5-17 years of age should accumulate an average of at least 60 minutes per day and up to several hours of at least moderate intensity physical activity. Some of the health benefits can be achieved through an average of 30 minutes per day. *[Level 2, Grade A]*. 2) More vigorous intensity activities should be incorporated or added when possible, including activities that strengthen muscle and bone *[Level 3, Grade B]*. 3) Aerobic activities should make up the majority of the physical activity. Muscle and bone strengthening activities should be incorporated on at least 3 days of the week *[Level 2, Grade A]*.

## Background

Canada's first set of physical activity guidelines for children and youth were introduced in 2002 [1,2]. The basic recommendation within these guidelines was that children and youth, independent of their current physical activity level, should *increase *the time they spend on moderate-to-vigorous intensity physical activity by 30 minutes per day, and over a 5 month period progress to adding an additional 90 minutes of daily physical activity. Recently, a narrative literature review was conduced to provide an update on the evidence related to the biological and psycho-social health benefits of physical activity in school-aged children and youth which has accumulated since the publication of Canada's guidelines [3]. This narrative review explored whether Canada's physical activity guidelines for children and youth are appropriate, and made recommendations as to how the guidelines could be modified to reflect current knowledge.

Several other narrative reviews have examined the relation between physical activity and health in school aged children, a small sample of which are referenced here [4-8]. Although informative, narrative reviews have severe limitations. First and foremost, it is uncertain as to whether all of the relevant scientific evidence has been examined. The authors of a narrative review may be exclusive with the materials they review, and these materials may have been selected and interpreted in a biased manner. Thus, the reader is faced with uncertainty and doubt when interpreting a narrative review. The reader may be better served when the choices made in the review are explicit, transparent, clearly stated, and reproducible. This can be achieved through a systematic review. Systematic reviews attempt to reduce reviewer bias through the use of objective, reproducible criteria to select relevant publications, to synthesize and critically appraise the findings from these publications, and to employ defined evidence-based criteria when formulating recommendations [9].

The purpose of this report was to: 1) perform a systematic review of the evidence informing the relation between physical activity and health in school-aged children and youth, defined here as those aged 5-17 years; and 2) make recommendations on the appropriate volume, intensity, and type of physical activity for minimal and optimal health benefits in school-aged children and youth. A previously developed evaluation system was used to set the level of evidence and grade for the recommendations. This report was part of a much larger project around Canada's physical activity guidelines, and comparable systematic reviews for adults [10] and older adults [11] have also been published in the journal. Additional details on the scope and purpose of the larger project [12] and the interpretation of the recommendations from an independent expert panel [13] can also be found elsewhere in the journal.

### Overview of existing physical activity guidelines for children and youth

Before conducting the systematic review, this paper provides a brief overview on existing physical activity guidelines for school-aged children, as well as an explanation of the scientific evidence that informed the guideline development process.

The publication of Canada's physical activity guidelines for children and youth in 2002 represented a joint effort of the Canadian Society for Exercise Physiology and Health Canada. Two sets of guidelines were published, one for children aged 6 to 9 years [2] and a second for youth aged 10 to14 years [1]. In addition to the physical activity guides, which highlighted the recommended physical activity levels for these two age groups, a number of other promotional and educational packages were developed, including family booklets [14,15], teacher booklets [16,17], as well as physical activity magazines for children [18] and youth [19].

The key recommendations within Canada's child and youth physical activity guides are:

1) Increase the time currently spent on physical activity by 30 minutes per day, and progress over approximately 5 months to 90 minutes more per day.

2) Physical activity can be accumulated throughout the day in periods of at least 5 to 10 minutes.

3) The 90 minute increase in physical activity should include 60 minutes of moderate activity (e.g., brisk walking, skating, bicycle riding) and 30 minutes of vigorous activity (e.g., running, basketball, soccer).

4) Participate in different types of physical activities - endurance, flexibility, and strength - to achieve the best health results.

5) Reduce non-active time spent on watching television and videos, playing computer games, and surfing the Internet. Start with 30 minutes less of such activities per day and progress over the course of approximately 5 months to 90 minutes less per day.

Many other countries and organizations have developed physical activity recommendations for school-aged children and youth, as recently summarized [3]. With few exceptions, these countries and organizations recommend that children and youth participate in at least 60 minutes of moderate-to-vigorous intensity physical activity on a daily basis. One of these recommendations was published in 2005 as part of a systematic review that linked physical activity to several health and behavioural outcomes in school-aged children and youth [20]. This systematic review was sponsored by the U.S. Centers for Disease Control and Prevention (CDC) and was developed by a multidisciplinary expert panel. The expert panel considered over 850 articles published in 2004 or earlier, identified by computerized database searches and by searching the bibliographies of the panellists' own libraries [20]. Based on conceptual definitions and inclusion and exclusion criteria developed by the panel, participants systematically evaluated relevant articles (primarily intervention studies) for each of the 14 health and behavioural outcomes considered. On the basis of their reviews, the panel provided a summary of the evidence for strength (strong, >60% of studies reviewed; moderate, 30-59% of studies reviewed; and weak, <30% of studies reviewed) and the direction (positive, null, or negative) of the effects of physical activity on each of the health and behavioural outcomes. The strength of evidence was judged from the statistical significance of the outcomes; it did not include other factors usually considered in systematic review, such as the effect sizes of physical activity and the quality and types of studies.

The expert panel reached the following conclusions: (i) Evidence-based data are *strong *to conclude that physical activity has beneficial effects on adiposity (within overweight and obese youth), musculoskeletal health and fitness, and several components of cardiovascular health. (ii) Evidence-based data are *adequate *to conclude that physical activity has beneficial effects on adiposity levels in those with a normal body weight, on blood pressure in normotensive youth, on plasma lipid and lipoproteins levels, on non-traditional cardiovascular risk factors (inflammatory markers, endothelial function and heart rate variability), and on several components of mental health (self-concept, anxiety and depression) [20]. A summary of evidence concerning the health outcomes examined by the expert panel is shown in Table 1 [Additional file 1]. The amount, intensity, and type of physical activity required to achieve the result, when clear, is also shown in the table.

In 2008 a second systematic review of literature examining the relation between physical activity and key fitness and health outcomes within school-aged children and youth was published. This systematic review was part of the "Physical Activity Guidelines for Americans" project that was undertaken by the Unites States Department of Health and Human Services [21]. Unlike the 2005 CDC sponsored systematic review that focused on intervention studies, the 2008 review considered both observational and experimental studies. The 2008 systematic review concluded that few studies have provided data on the dose-response relation between physical activity and various health and fitness outcomes in children and youth. However, substantial data indicate that health and fitness benefits will occur in most children and youth who participate in 60 or more minutes of moderate-to-vigorous physical activity on a daily basis. For children and youth to gain comprehensive health benefits they need to participate in the following types of physical activity on 3 or more days per week: vigorous aerobic exercise, resistance exercise, and weight-loading activities.

Although informative, the recommendations made within the 2005 and 2008 systematic reviews did not include a level of evidence or grade, which are now becoming a routine part of evidence based reviews. The level of evidence helps inform the reader about the strength of evidence that informed the recommendation. The grade considers the harms and benefits of implementing the intervention, and informs the reader about whether an intervention should be implemented.

### Questions addressed in systematic review

The following questions were addressed in this systematic review:

1) *How much (volume) physical activity is needed for minimal and optimal health benefits in school-aged children and youth? *To address this question careful consideration was given to whether dose-response relations existed between physical activity and fitness with the various health outcomes, and if so, the pattern of these relations (e.g., linear, or curvilinear relations with large improvements in health occurring with limited increases in physical activity at the low end of the physical activity scale, or curvilinear relations with small improvements in health occurring with increases in physical activity at the low end of the physical activity scale).

2) *What types of activity are needed to produce health benefits*? Specific consideration was given to what types of activity (aerobic, resistance, etc.) influenced the different health outcomes, and whether more than one type of activity would be needed for overall health and well-being.

3) *What is the appropriate physical activity intensity*? Attention was given to the intensity of physical activity measured (observational studies) or prescribed (experimental studies). An underlying assumption was that children and youth would prefer lower intensity activities over higher intensity activities. Therefore, for higher intensity activities to be recommended over lower intensity activities there would need to be either: i) no evidence that low intensity activities were beneficial for health and evidence that higher intensity activities impacted health in a favorable manner, or ii) clear evidence that higher intensity activities impacted the health outcomes to a greater extent than lower intensity activities.

4) *Do the effects of physical activity on health in school-aged children and youth vary by sex and/or age*? Results were examined to see if: i) the moderating effects of sex and/or age on the relations between physical activity and health were explored, and if not, iii) whether there were consistent patterns across studies (either statistically or in order of magnitude) that were suggestive of sex or age differences.

## Methods

### Eligibility criteria

This systematic review was limited to key indicators of different health outcomes known to be related to physical activity in school-aged children and youth. Decisions on what health outcomes to include in the systematic review were made by examining what outcomes were studied in previously conducted reviews of this nature [20,21] and in consultation with the Steering Committee for the Canadian Physical Activity Guidelines project. These key indicators consisted of:

1) High blood cholesterol, high blood pressure, and markers of the metabolic syndrome as a measure of cardiometabolic risk

2) Overweight/obesity as a measure of adiposity

3) Low bone density as a measure of skeletal health

4) Depression as a measure of mental health

5) Injuries as a negative health outcome of physical activity

We recognized that although cardiorespiratory and musculoskeletal fitness are partially genetic in origin, they are in large measure a reflection of physical activity participation in recent weeks and months [22]. Therefore, the systematic review also included studies that examined the relation between fitness and health. For our purposes, fitness was assumed to be a proxy measure of physical activity. Any studies evaluating the relationship between physical activity or fitness and one or more of the key health outcomes listed above within school-aged children and youth were eligible for inclusion.

In consultation with the Steering Committee of the Canadian Physical Activity Guidelines and Measurement Project and the authors who were completing the adult and older adult systematic reviews, a decision was made to limit the pediatric systematic review to: 1) studies examining the key health indicators above, and 2) for observational studies, the outcomes must have been measured in a dichotomous (yes or no) manner and presented as prevalences or ratio scores (odds ratio, relative risk, hazard ratio). This decision was made for three reasons: (i) to help ensure that the systematic review would be manageable in size and scope for a single research team to complete in a timely manner, (ii) to eliminate many of the observational studies with small sample sizes, and (iii) to ensure that the health outcomes, at least for the observational studies, were presented in a reasonably consistent pattern from study to study. This helped us to make comparisons between studies and to characterize the magnitude of effect for physical activity.

To further illustrate why the aforementioned limitations were put in place, consider the following. Within children and youth physical activity has been related to over two dozen different health outcomes. For adiposity alone, several adiposity measures have been considered including body weight, BMI, several skinfold and circumference measures, total body fat, and several specific body fat depots. Preliminary literature searches on adiposity - as measured using both continuous (e.g., body weight, BMI, visceral fat, skinfolds) and categorical (overweight/obese vs. normal weight) outcomes to capture the measures indicated above, revealed over 15,000 published papers. The results from these papers were presented in several formats including comparison of group means for continuous adiposity measures according to physical activity level, relations between continuous adiposity and physical activity measures which were presented in a variety of ways (e.g., r values, regression coefficients); comparison of group means for physical activity according to adiposity status; ratio scores (odds ratios, relative risks, hazard ratios) for the prediction of obesity status according to physical activity level; comparison of the prevalence of obesity according to physical activity level; etc. Without employing some criteria to limit the types of measures and outcomes, it would have been virtually impossible to synthesize the results from these studies.

Cross-sectional studies, case-control studies, cohort studies (prospective and retrospective) and intervention studies (including randomized and quasi experimental designs) were eligible for inclusion in the systematic review. Only published, English language studies including human participants were included. To be included studies had to be limited to school-aged children and youth between 5-17 years of age, or present data specifically for a subgroup of participants within this age range.

For the observational studies, there were no limitations placed on the form of physical activity (e.g., questionnaire, activity diary, pedometer, accelerometer) or fitness (cardiorespiratory or musculoskeletal fitness) measurements. For intervention studies, all cardiorespriatory and/or musculoskeletal based interventions were eligible for inclusion. Intervention studies were excluded if they included a dietary (e.g., caloric restriction) or other behavioral risk factor component (e.g., smoking cessation) that may have independently affected the health outcomes and subsequently made it impossible to distinguish the independent effect of the physical activity portion of the intervention.

### Search strategy

Literature searches were conducted in MEDLINE (1950-January 2008, OVID Interface), EMBASE (1980-January 2008, OVID Interface), CINAHL (1982-January 2008, OVID Interface), PsycINFO (1967-January 2008, OVID Interface), all Evidence-Based Medicine Reviews (1991-January 2008, OVID Interface), and SPORTDiscus (up to January 2008, EBSCO Interface).

The electronic search strategies were executed by a single researcher (AB) under direction of the primary author (IJ). They were not restricted by publication type or study design; however, they were limited to human participants and English language. The following string of search terms were used for each of the study outcomes to identify physical activity related papers conducted within the age group of interest: ('physical activity' OR 'fitness' OR 'exercise' OR 'energy expenditure') AND ('child' OR 'adolescent' OR 'youth' OR 'juvenile'). The following search terms were added (e.g., AND) for the cholesterol search: ('high cholesterol' OR 'hypercholesterolemia' OR 'hyperlipidemia' OR 'dyslipidemia'). For hypertension the following search terms were added: ('high blood pressure' OR 'hypertension'). For metabolic syndrome the following search terms added: ('metabolic syndrome' OR 'syndrome X' OR 'deadly quartet' OR 'plurimetabolic syndrome' OR 'insulin resistance' OR 'insulin resistant'). For obesity the following search terms were added: ('obese' OR 'obesity' OR 'overweight'). For low bone mineral density the following search terms were added: ('bone density' OR 'bone strength' OR 'bone mass' OR 'bone mineral density'). For depression the following search terms were added: ('depression' OR 'mood disorder'). For injuries the following search terms were added: ('injury' OR 'injuries').

**Figure 1:**
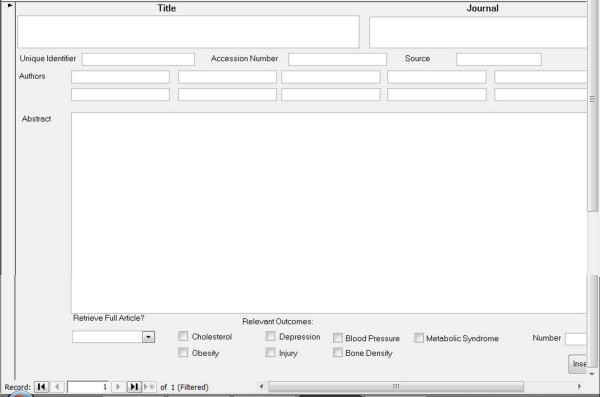
**Copy of electronic abstract review form**.

A total of 42 electronic searches were performed (7 health outcomes × 6 search engines) and the information from each search was saved as a text file that included all of the retrieved citations. Using SAS software version 9 (SAS Institute, Carry, NC), the text files were separated back into individual citations and exported into a Microsoft Access database. The database included the following information for each citation: unique identifier for the database, paper title, authors, journal name, volume and issue number, page numbers, and the abstract. See Figure 1 for an illustration of the Microsoft Access database form. Within the Microsoft Access database, duplicate citations - those citations that were identified in more than one of the search engines and/or for more than one of the health outcomes - were identified by a match of the title and were removed using automated procedures.

### Screening of citations

After duplicate citations were removed from the Access database, the abstract of each citation was reviewed by a single reviewer to determine if it should be included within the systematic review. The full-text articles of all potentially relevant citations were obtained, and saved as Adobe-PDF files that were linked to the Access database. Whenever it was uncertain as to whether a citation was appropriate, the full-text copy was obtained. After the first reviewer screened the database, the citations that were deemed ineligible were reviewed by a second reviewer to determine if any potentially relevant citations were missed, and full-text copies of these citations were also obtained. Copies of all of the full-text articles were then reviewed by the two reviewers for inclusion criteria; if uncertain as whether or not to include an article, the article in question was reviewed again until a final decision was made.

### Data abstraction

**Figure 2:**
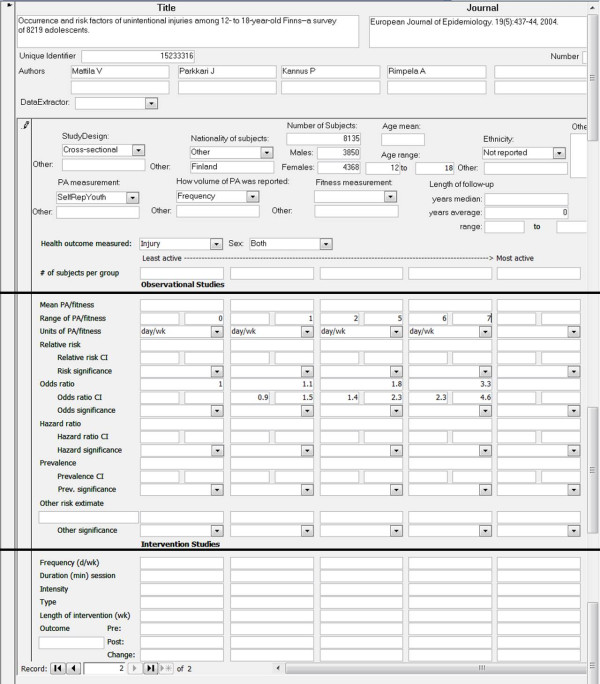
**Copy of electronic database abstraction form**.

A single reviewer (AL) abstracted data from all eligible full-text citations using an electronic data abstraction form. Refer to Figure 2 for an illustration of electronic data abstraction form. The data abstraction was completed in a second Microsoft Access Database, which was linked to the first Microsoft Access Database using a unique identifier. The abstracted data included information on the study design, participants, details of the physical activity (or fitness) measures or interventions, and key findings. After data abstraction was completed, the information was checked by a second reviewer (IJ) and corrected when necessary.

### Assigning levels of evidence and formulation of recommendations

The goal was to use a rigorous, evidence-based approach to develop levels of evidence on the relation between physical activity and health in school-aged children that could be used to formulate recommendations for the specific volume, intensity, and type of physical activity needed. At present there is no universally accepted method for formulating evidence-based recommendations. In consultation with the Steering Committee for this project and the authors performing the systematic reviews in adults and older adults, we chose to use the process that was recently employed for the development of Canada's obesity prevention and management guidelines [23]. Within this system, the level of evidence for a recommendation is based on an objective appraisal of the literature according to a pre-specified scale as reflected by the study designs and quality. As shown in Table 2 [Additional file 2], the level of evidence can range from 1 (highest) to 4 (lowest). The grade for a recommendation reflects the level of evidence and several additional features, including: benefits and risks of physical activity participation, magnitude of the effects, cost of the intervention, and value of an intervention to an individual or population. As indicated in Table 3 [Additional file 3], the grade for the recommendation may be an A, B, or C. Note that while the level of evidence assigned is not necessarily linked to the corresponding grade, a high grade is less likely in the setting of low-quality of evidence.

Note that the level of evidence in the aforementioned grading system is based in part on the quality of the studies. This grading was particularly relevant for experimental studies wherein the level of evidence would change from Level 1 to Level 2 based on whether or not the randomized controlled trials (RCTs) have important limitations. A single investigator (IJ) assessed the quality of the RCTs included in this systematic review using the validated checklist developed by Downs and Black [24]. This 27-item checklist assess the quality of reporting (e.g., are the interventions of interest clearly described, have all the adverse events that may be a consequence of the intervention been reported), external validity (e.g., were the subjects representative of the population), internal validity (e.g., was an attempt made to blind those measuring the outcome, were the outcome measures accurate), selection bias (e.g., were the study subjects randomized, was randomization assignment concealed until recruitment was complete), and statistical power.

To evaluate the magnitude of effect of physical activity on the various health outcomes examined, in addition to statistical significance, the following criteria were applied to evaluate the strength of the ratio scores (odds ratio, relative risk, hazard ratio) for the observational studies. For positive associations 1.01-1.50 = weak association, 1.51-3.00 = moderate association, and 3.01 or higher = strong association. For negative associations: 0.71-0.99 = weak association, 0.41-0.70 = moderate association, 0.00-0.40 = strong association [25]. For the experimental studies, measures of effect were calculated based on Cohen's d, which was calculated as the difference between the pre- and post-treatment mean within a given treatment group divided by the average of the standard deviation of the pre- and post-treatment means [26]. Cohen's d effect measures ≥ 0.49 were considered to be weak, values ranging from 0.50-0.79 were considered to be moderate, and values ≥ 0.80 were considered to be strong [26]. Note that several experimental studies did not report the information required to calculate Cohen's d, and for these studies effect measures have not been presented.

When possible (e.g., at least 4 studies) we performed meta-analyses to calculate summary odds ratio and Cohen's d effect size measures for the observational and experimental studies, respectively [27]. These summary estimates represent a weighted average of the estimates provided in the various studies included in the meta-analysis. These meta-analyses were performed separately for each health outcomes, separately for observational and experimental studies, and separately based on type of physical activity measurement or exercise modality prescribed.

## Results

### Literature review

**Figure 3:**
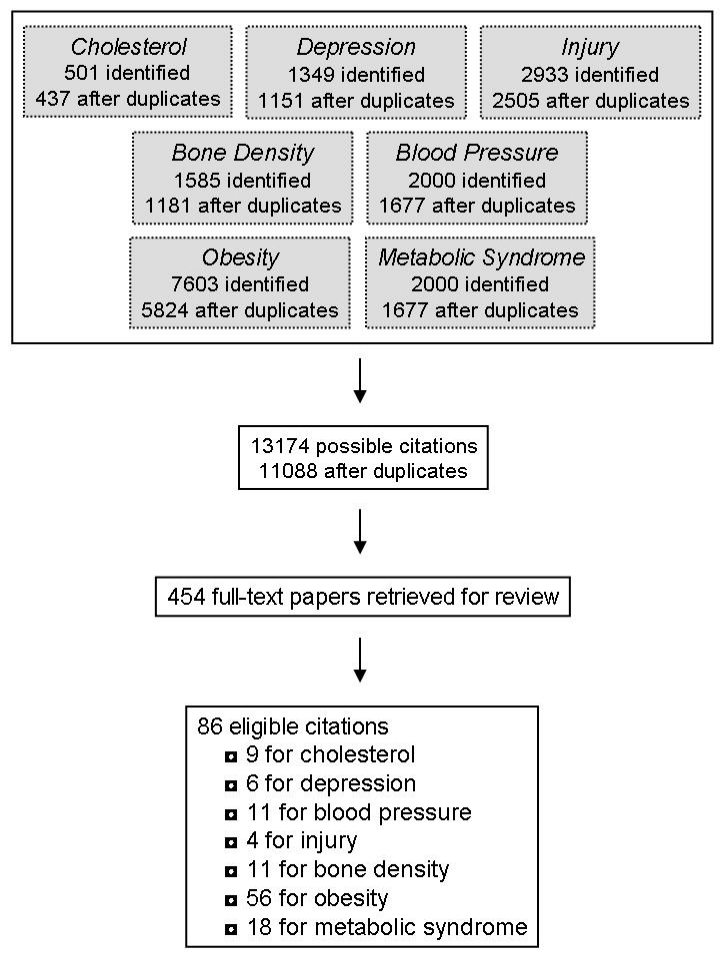
**Flow of articles through the systematic review**.

The flow of citations through the systematic review process is shown in Figure 3. For each of the 7 health outcomes, several citations were retrieved in more than one of the 6 search engines. After removing duplicates, a total of 437 citations were identified for cholesterol, 1151 for depression, 2505 for injury, 1181 for bone density, 1677 for blood pressure, 5824 for obesity, and 1677 for the metabolic syndrome. Thus, the grand total was 13174. Many of these 13174 citations were retrieved for 2 or more health outcomes, and after removing these duplicates there was a total of 11,088 unique citations. After the titles and abstracts of these 11,088 citations were reviewed, full-text copies of 454 potentially relevant citations were obtained and reviewed. Of these 454 citations, 86 unique citations passed the eligibility criteria and were included in the systematic review. Several of these 86 citations included results for 2 or more of the 7 relevant health outcomes.

### Cholesterol and blood lipids

A total of 9 articles examining blood lipids and lipoproteins met the inclusion criteria. Only one of these studies was observational in nature [28]. This cross-sectional study was conducted on a representative sample (n = 3110) of 12-19 year old American adolescents and measured cardiorespiratory fitness using a submaximal treadmill test. The results indicated that unfit girls, defined as the lowest 20% fit, were 1.89 (95% confidence interval: 1.12-3.17) times more likely to have hypercholesterolemia and 1.03 (0.74-1.43) times more likely to have a low HDL-cholesterol by comparison to moderately and high fit girls. Unfit boys were 3.68 (2.55-5.31) times more likely to have hypercholesterolemia and 1.25 (0.79-1.95) times more likely to have a low HDL-cholesterol by comparison to moderately and high fit boys.

A total of 8 experimental studies (6 RCT, 2 non-randomized) examined the effect of exercise interventions on changes in blood lipids and lipoproteins, as summarized in Table 4 [Additional file 4] [29-36]. For the most part, these studies were limited to children and youth with high cholesterol levels [30] or obesity [29,32,34,36] at baseline. The sample sizes were quite small and only 2 of these interventions included more than 37 participants [32,36]. The interventions ranged from 6 to 24 weeks in duration and included anywhere from 1 to 4 hours per week (9-34 minutes per day on average) of prescribed exercise. Six of the 8 exercise programs included various forms of moderate-to-vigorous physical activity as explained in the methods sections of the papers.

The results from these intervention studies were mixed. The 5 studies that were based on aerobic exercise alone observed significant improvements in at least one lipid/lipoprotein variable. The summary effects size measures (95% confidence interval) for the aerobic exercise interventions were -3.03 (-3.22, -2.84) for triglycerides and 0.26 (0.03, 0.49) for HDL-cholesterol. The interventions that were based on resistance training [33] and circuit training [34] reported small and/or insignificant changes for all of the lipid/lipoprotein variables examined, and the effect sizes within these studies tended to be quite small (eg, <0.5). Not surprisingly, the interventions that produced significant changes were also based on the studies that employed the largest sample sizes. This suggests that many of the studies were underpowered.

Due to the design of these interventions (eg, only one dose of exercise prescribed in a given study), the nature of the dose-response relation between exercise and blood lipids in children and youth remains unclear. Furthermore, the interventions that produced favorable effects on blood lipids did not tend to prescribe higher volumes or intensities of exercise by comparison to the interventions that did not produce significant changes. The favorable interventions were, however, based on 'high risk' participants, implying that low volumes of moderate-to-vigorous exercise may be beneficial for youngsters at the greatest risk.

The effects of age and sex have not been adequately addressed in the existing literature. Thus, conclusions cannot be made on the moderating effects of these demographic characteristics on the relation between physical activity and blood lipids in school-aged children and youth.

### High blood pressure

A total of 11 articles examining high blood pressure met the systematic review inclusion criteria. Three of these studies were observational in nature (2 cross-sectional, one prospective cohort) (Table 5) [Additional file 5] [28,37,38]. Of these 3 studies, one relied on self-reported measures of physical activity [37] and the remaining two measured cardiorespiratory fitness [28,38]. Within all 3 observational studies the relations between physical activity or fitness with hypertension were weak in magnitude (e.g., odds ratios <1.5), and in one case [28] was insignificant. Only one study examined more than 2 levels of physical activity or fitness (e.g., compared risk estimates across at least 3 groups), and thus was able to provide some insight into the dose-response relation. Within that study only participants within the least fit quartile were more likely to have hypertension relative to participants in the most fit quartile, a finding that was consistent in boys and girls [38].

Eight experimental studies, 4 of which were RCTs, examined the influence of exercise interventions on changes in blood pressure (Table 6) [Additional file 6] [29,33,34,39-43]. Most of these studies were limited to children and youth with high blood pressure [39-42] or obesity [29,34]. The sample sizes were quite small; only one of these studies included more than 37 participants [42]. The interventions ranged from 4 to 25 weeks in duration. With one exception [43], the interventions included between 60 to 180 minutes/week of prescribed exercise. This equates to 9 to 30 minutes/day when averaged over a week.

Despite the small sample sizes, the results from these intervention studies were positive with reports of significant reductions in systolic blood pressure in response to aerobic exercise training, with effect sizes that all tended to be large (>0.80) [29,39,41-43]. Two of the aerobic based interventions also reported significant reductions (~6% to 11%) in diastolic blood pressure [34,39]. The summary effect size measures for the aerobic exercise interventions were -1.39 (-2.53, -0.24) for systolic blood pressure and -0.39 (-1.72, 0.93) for diastolic blood pressure. Unlike the aerobic-based exercise programs, only two of the four studies that employed other training modalities, such as resistance exercise, reported a significant effect on blood pressure [33,40], with small to modest effect sizes being observed. The summary effect size measures for the non-aerobic exercise interventions were -0.61 (-2.27, 1.05) for systolic blood pressure and -0.51 (-2.18, 1.06) for diastolic blood pressure.

Because the aerobic exercise intervention studies prescribed similar volumes and intensities of exercise, and because they found comparable reductions in blood pressure, the effects of the volume and intensity (moderate vs. vigorous) of exercise on blood pressure remain unclear. Due to limited variations in the age of the participants in these studies, the effects of age on the relation between exercise and blood pressure remains unclear. Most of the studies included both males and females, suggesting that aerobic exercise is effective at controlling blood pressure within box sexes.

### Metabolic syndrome

The metabolic syndrome has received considerable research attention in recent years in both adults and youngsters. Sixteen articles examining the metabolic syndrome met the inclusion criteria. The metabolic syndrome components (e.g., abdominal obesity, triglycerides, insulin, HDL-cholesterol, inflammatory markers, etc.) and criteria (e.g., cut-points used to define high-risk values) employed in these studies varied considerably. Eight of the 17 identified studies were observational in nature (7 cross-sectional, 1 prospective), as summarized in Table 7 [Additional file 7] [28,44-51]. Many of these observational studies examined large and heterogeneous samples of participants, suggesting that the findings are quite generalizable to the general population. Of the 3 cross-sectional studies that employed self-reported measures of physical activity, the reported relations with the metabolic syndrome were either weak or modest in strength, and all were non-significant [45,48,49]. The summary odds ratio for the least active group relative to the most active group in these 3 studies was 1.68 (95% confidence interval: 1.22, 2.31). By comparison, the study that used accelerometers to measure physical activity in an objective manner [51] and the 4 studies that used direct measures of cardiorespiratory fitness [28,46,47,50] all reported strong and significant relations with the metabolic syndrome. The summary odds ratio for the least fit group relative to the most fit group in the 4 studies that measured fitness was 6.79 (95% confidence interval: 5.11, 9.03). Further examination of these later studies revealed clear dose-response relations; however, the nature (e.g., linear or curvilinear) of the dose-response relation is unclear. In addition, comparison of the risk estimates in males and females suggests that the relation between physical activity and fitness with the metabolic syndrome is stronger in males. The influence of age on these relations remains uncertain.

Eight experimental studies, 5 of which were RCTs, examined the effect of exercise interventions on changes in markers of the metabolic syndrome, primarily in the form of fasting insulin and insulin resistance (Table 8) [Additional file 8] [32-34,36,52-55]. All but one of these studies was conducted in an overweight/obese sample [33]. The number of participants included in these studies was modest, with all but a single study being limited to 52 participants or less [36]. The exercise interventions ranged from 6 to 40 weeks in duration and included anywhere from 80 to 200 minutes per week (10-30 minutes per average day) of prescribed exercise. About half of the exercise programs were aerobic in nature.

The results from these 8 studies were mixed (Table 8). All of the 4 interventions that focused on aerobic exercise observed significant improvements in at least one of the insulin variables examined. Conversely, only one of the four interventions that employed resistance or circuit training observed any meaningful improvements [54]. The summary effect size measures (95% confidence interval) for fasting insulin in the aerobic and resistance exercise interventions were -0.60 (-1.71, 0.50) and -0.31 (-0.82, 0.19), respectively. No intervention studies systematically considered the influence of the dose or intensity of exercise, or sex and age effects, on markers of the metabolic syndrome. More research is needed to address these issues.

### Overweight and obesity

The relation between physical activity and fitness with obesity in school-aged children and youth has been extensively studied. A total of 31 observational studies (24 cross-sectional, 3 prospective cohort, 2 case-control, 1 mixed) were retrieved that met the appropriate inclusion criteria, as summarized in Table 9 [Additional file 9] [56-86]. Overweight and obesity were classified using age- and gender-specific body mass index (BMI) criteria (e.g., BMI z-scores) in the majority of these observational studies. The majority of these studies assessed physical activity or sport participation using self- or parental-reported tools. These studies tended to report weak to modest relationships between physical activity and overweight/obesity, with many risk estimates being non-significant. Of the 25 available data points, the median odds ratio for overweight/obesity in the least active group relative to the most active group was 1.33. It is noteworthy that the studies that assessed moderate-to-vigorous intensity physical activities alone were more consistently and strongly related to obesity than the studies that included low intensity activities within the physical activity measure.

Four studies were identified that employed objective measures of physical activity, including one study that used pedometers [73] and 3 studies that used accelerometers [59,67,83]. These studies tended to report significant relations between physical activity with overweight/obesity that were strong in magnitude. Of the 8 available data points for cross-sectional findings, the median odds ratio was 3.79. An additional 4 studies measured the relation between cardiorespiratory fitness and obesity [75,76,82]. All of these studies reported significant relations between physical activity and fitness with overweight/obesity that were modest to strong in magnitude.

Several of the observational studies examining overweight and obesity presented analyses that were stratified by sex [56-58,60,64,67,71,73-75,77,79,81]. Although sex differences were rarely tested for using the appropriate statistical techniques, visual inspection of the risk estimates provided suggests that in 12 of the 14 studies the associations between physical activity and fitness with obesity were stronger in males than in females.

Many of the observational studies presented their results in a manner that permitted the dose-response relations with obesity to be examined [57,59,61,62,64,65,69,71,73,74,79,80,82,84]. From these studies it is apparent that a dose-response relation between physical activity and obesity exists. However, the pattern of this dose-response relation is unclear as some studies observed linear patterns and others observed curvilinear patterns.

In addition to the observational studies discussed above, 24 intervention studies, 17 of which were RCTs, examining changes in obesity measures were included in the systematic review (Table 10) [Additional file 10] [29,32,34-36,39,42,43,52-55,87-98]. It is important to note that in many of these studies the primary aim of the intervention was to improve other health measures (e.g., blood lipids, insulin resistance, and bone density) and not obesity measures *per se*. These studies examined several different measures of total (% fat, BMI, weight) and abdominal (waist circumference, trunk fat, visceral fat) adiposity. The studies ranged in length from 4 weeks to 2 years, with most being 4 to 6 months in duration. The amounts of exercise prescribed typically ranged from 2 to 3.5 hours per week, which averages out to 17 to 30 minutes per day. Half of the studies were limited to overweight and obese participants.

About 50% of the exercise interventions that were aerobic in nature observed significant changes in measures of BMI, total fat, and/or abdominal fat in response to training. Only 3 of the 17 studies that employed other training modalities (resistance training, circuit training, pilates, jumping exercises) observed significant improvements in measures of total fat, abdominal fat, or BMI in response to training. The effect sizes, even for the studies that found significant improvements, tended to be small (<0.50). For the interventions that were based on aerobic exercise, the summary effect size measures were -0.40 (-1.10, 0.31) for % body fat and -0.07 (-0.89, 0.75) for BMI. For the resistance exercise intervention, the summary effect size calculation for % body fat was -0.19 (-1.55, 1.18).

Variations in the effects of age, sex, and exercise dose on changes in obesity measures in response to exercise training have not been systematically addressed in the literature. Thus, no conclusions can be drawn on the potential moderating effects of these variables.

### Bone mineral density

Many observational studies have examined the relation between physical activity and *continuous *measures of bone mineral density such as bone mineral content values in grams, bone density values in g/cm^2^, and cortical bone area measures in cm^2 ^(see review [99]). However, no observational studies in the literature search met the systematic review criteria of predicting a low bone mineral density as a *dichotomous *outcome.

As summarized in Table 11 [Additional file 11], a total of 11 experimental studies examining changes in bone mineral density in response to exercise training were retrieved in the systematic review [55,88-94,100-103]. Two of these studies presented identical data on the same group of participants, and were therefore presented as a single study in the table [101,102]. The physical activity programs employed in these interventions typically consisted of moderate-to-high strain anaerobic activities such as impact resistance training, high impact weight bearing, and jumping. These programs were performed anywhere from 3 to 60 minutes in length on at least 2 or 3 days of the week, and lasted from a few months to 2 years in duration.

The results from these studies, although not undisputed, indicate that as little as 10 minutes of moderate-to-high impact activities performed on as little as 2 or 3 days of the week can have a modest effect on bone mineral density when combined with more general weight bearing aerobic activities that are also beneficial for cardiovascular risk factors and obesity prevention (e.g., jogging, play, etc.).

### Depression

Only 6 studies on depression and related symptoms met the inclusion criteria. Table 12 [Additional file 12] outlines the 3 observational studies [104-106]. These were all cross-sectional in design, used self-reported measures of physical activity, and reported small and insignificant [104,106] or modest [105] relations between physical activity and depression. Interestingly, within the later study the relation between physical activity and depression were more evident at a moderate intensity of physical activity than at a vigorous intensity of physical activity [105].

The 3 experimental studies that examined changes in depression [107-109], all of which were RCTs based on aerobic exercise, are outlined in Table 13 [Additional file 13]. The volume of exercise prescribed in these studies was very modest (60 to 90 minutes per week). All three of these studies observed significant improvements in at least one depressive symptom measure in response to 8 to 12 week exercise programs. The effect sizes were small to modest in these studies, with very broad 95% confidence intervals. One of the studies included both high intensity and moderate intensity exercise programs, and only the high intensity program resulted in significant improvements in depression scores in comparison to the control group, which performed flexibility exercises [108].

### Injuries

Injuries are a leading cause of disability and mortality in young people. It has been reported that approximately 50% of medically treated injuries within 6^th ^to 10^th ^grade Canadian youth occur during physical activity [110]. Thus, it is not surprising that there is an extensive literature on physical activity and injuries in the pediatric population (see review [111]). However, most of the published information is limited to groups of participants that have all been injured or groups of participants comprised entirely of athletes (eg, football players, ballet dancers).

Only 3 articles examining injury met the inclusion criteria for this systematic review [112-114]. These studies were all cross-sectional in nature and relied on self- or parental-reported measures of physical activity and sports participation (Table 14) [Additional file 14]. These studies examined medically treated injuries; however, limited or no information on the severity of and long-term recovery from these injuries was presented. All 3 of the studies reported higher rates of injury in physically active children and youth compared with inactive children and youth. Furthermore, within all 3 of the papers there was clear evidence of a dose-response relation between physical activity participation and the likelihood of injury. That is, as the physical activity level increased, the likelihood of injury increased in a graded fashion. One study assessed vigorous sports, and within that study the risk estimates for injury within the most active group would be considered high [114]. Conversely the risk estimates for injury were modest within the 2 studies that measured moderate-to-vigorous intensity activities [112,113]. The quality of the evidence for the injury outcome, which is based on cross-sectional studies, is limited as cross-sectional research only provides a low level of evidence. Follow-up (incidence) studies that also take into consideration the volume of sports participation would provide a more powerful level of evidence.

### Quality assessment of RCTs

The RCTs that are listed within the summary tables contained several significant limitations. The study samples were small, and non-representative. Although few of the studies addressed the issue of statistical power, the lack of power was clearly an issue. Specifically, for a number of the health outcomes, the RCTs in which significant findings were observed were also the RCTs with the largest sample sizes. Almost without exception, the RCTs included in the systematic review did not report adverse events for the physical activity interventions (e.g., injuries), provided little or no detail on the drop-outs, and did not perform intent-to-treat analyses. Given the consistency of these limitations across studies, Level 2 was the highest level that could be assigned to any of the recommendations.

## Discussion

### Recommendations based on systematic review

*Recommendation #1*

*Children and youth 5-17 years of age should accumulate an average of at least 60 minutes per day and up to several hours of at least moderate intensity physical activity. Some of the health benefits can be achieved through an average of 30 minutes per day. [Level 2, Grade A]*

There is strong and consistent evidence based on experimental studies for several health outcomes that participating in as little as 2 or 3 hours of moderate-to-vigorously intense physical activity per week is associated with health benefits. Evidence from observational studies also demonstrates dose-response relations between physical activity and health, with differences in health risk between the least active (or fit) and the second least active (of fit) groups. Thus, it would seem appropriate to set minimal physical activity targets that reflect a low level of physical activity (see Recommendation #1). Furthermore, the current recommendation of 90 minutes more per day (Canadian) or 60 minutes per day (US, UK, Australian) may be quite intimidating, particularly for children and youth who are very inactive. From a behaviour modification perspective, having a target that seems out of reach may actually undermine physical activity participation [115].

That being said, with the exception of injuries, the dose-response evidence from observational studies for several health outcomes suggests that more physical activity will be better, and that additional health benefits can still be achieved at the higher end of the physical activity spectrum. Therefore, it would also seem appropriate to set higher physical activity targets (60 minutes and up to several hours) that would elicit more pronounced health benefits for those children and youth who are already somewhat active (see Recommendation #1). This approach is consistent with recommendations made by the U.S. National Association for Sports and Physical Education[116] and the Australia Department of Health and Ageing [117], both of whom have recommended that children and youth participate in at least 60 minutes, and up to several hours, of moderate to vigorous intensity physical activity every day.

This type of dual message provided in Recommendation #1 will hopefully encourage children and youth who are very inactive to engage in at least a modest amount of physical activity, while at the same time encourage moderately active children and youth to achieve even greater benefits by becoming more active. The minimal and optimal doses of physical activity required for good health in children and youth remain unclear, and more carefully conducted dose-responses studies are warranted in the pediatric age range.

Previous physical activity recommendations and guidelines for school-aged children and youth indicate that a high volume of physical activity needs to be performed everyday. The need for children and youth to engage in physical activity on a daily basis to maintain good health was not supported by the evidence reviewed here. In other words, it is unknown as to whether a child who accumulates 7 hours of activity over the week, with one hour being performed on each day, would have any greater health benefits than a child who accumulates 7 hours of activity over the week, with different amounts of activity being performed each day (including some days with no activity). Thus, the recommendation made in this systematic review calls for an "average" of at least 60 minutes per day instead of at least 60 minutes everyday. Future studies need to address whether a "days per week" recommendation is warranted. In addition, future studies within children and youth should consider whether the daily physical activity needs to be accumulated in bouts of at least a few minutes in duration (eg, 5 or 10 minutes). Most children accumulate the majority of their physical activity in a very sporadic manner (eg, a couple of minutes here and there), and new evidence suggests that this sporadic pattern of activity may not be as beneficial as bouts of activity that last at least 5 minutes in length [118].

Given the positive effect of physical activity on 6 of the 7 health outcomes examined, including observations from several large and diverse samples, this Recommendation was assigned a Grade A.

*Recommendation #2*

*More vigorous intensity activities should be incorporated or added when possible, including activities that strengthen muscle and bone. [Level 3, Grade B]*. 

Moderate intensity activity in children and youth has been defined in a variety of ways, depending on the method chosen to measure physical activity. The lower threshold of moderate intensity activity is usually defined as 4 METS (4 × resting metabolic rate), although it is not uncommon for investigators to use 3 METS. In general, the lower threshold of vigorous intensity activity is usually defined as 7 METS (7 × resting metabolic rate) in children.

The majority of observational studies have focused on measuring moderate-to-vigorous intensity physical activity. Furthermore, the relations between overall physical activity (including low intensity activities) and obesity do not appear to be as strong or consistent as the relations between moderate-to-vigorous intensity activity and obesity. In addition, the intervention studies included within this systematic review almost exclusively prescribed physical activity of at least a moderate intensity. Thus, while it is clear that moderate and vigorous intensity activities are associated with many health benefits, the same is not true for low intensity activity. Therefore, Recommendation #1 indicates that the physical activity should be of at least a moderate intensity. More consideration on the impact of low intensity activities on health should be given in future studies.

The next question to address is whether vigorous intensity activities provide benefits above and beyond that of moderate intensity activities. Regrettably, few studies have systematically addressed this question. The available information suggests that vigorous intensity activities provide additional health benefits beyond modest intensity activities. Furthermore, many of the experimental studies that observed significant changes in the health variables examined prescribed exercise that would fall within the vigorous intensity or upper-end of the moderate intensity range. Recommendation #2, therefore, suggests that vigorous intensity activities should be included when possible. This recommendation was assigned a lower level of evidence (Level 3) because of the limited amount of evidence and the inconsistency in the evidence that is available. This recommendation was given a lower grade (Grade C) because of the potential increase in injury risk associated with more vigorous intensity activities and sports. However, the injury data is weak and future studies, particularly intervention studies, should examine and report on injuries associated with physical activity in children.

*Recommendation #3*

*Aerobic activities should make up the majority of the physical activity. Muscle and bone strengthening activities should be incorporated on at least 3 days of the week. [Level 2, Grade A]*. 

Many of the health outcomes examined, particularly obesity and the cardiometabolic health measures, responded almost exclusively to aerobic exercise interventions. It is also likely that most of the activity that was captured in the observational studies was aerobic in nature. Recommendation #3 therefore suggests that physical activity should focus on aerobic activities. However, bone health was more favorably affected by modest amounts of resistance training and other high-impact activities (jumping) that were performed on at least 2 or 3 days of the week. Thus, this recommendation indicates that a small amount of bone strengthening activities should be incorporated.

### Limitations

This systematic review has several limitations, many of which related to practical issues around conducting the study (e.g., budgetary, human resource, and time constraints). First, because we did not include unpublished studies and studies that were published in a language other than English, and because we did not perform an extensive cross-referencing of the references lists from the papers that were retrieved in the electronic databases, several relevant papers may be been excluded. Second, the review was limited to 7 health outcomes and did not include several other outcomes that may be relevant for children and youth such as academic performance, emerging cardiometabolic risk factors (e.g., endothelial function, inflammatory markers), risky and aggressive behaviours (e.g., substance use and abuse, bullying and fighting), and measures of mental health and well-being outside of depression. Third, a large percentage of observational studies in the area were excluded because they did not report their findings in a dichotomous manner. Together, these limitations may have biased the Results and Recommendations that were made. Nonetheless, despite these limitations and the differences in methodology employed, the recommendations made here are remarkably comparable to the recommendations for children and youth that were part of the recently completed "Physical Activity Guidelines for Americans" project [21]. The reader is referred to the Expert Panel report for a more comprehensive discussion of the limitations of this systematic review [13].

## Conclusion

In summary, the findings of this systematic review confirm that physical activity is associated with numerous health benefits in school-aged children and youth. The dose-response relations between physical activity and health that were observed in several observational studies suggest that the more physical activity, the greater the health benefit. However, the results from several experimental studies suggested that even modest amounts of physical activity can have tremendous health benefits in high-risk youngsters (e.g., obese, high blood pressure). To achieve substantive health benefits, the physical activity should be of at least a moderate intensity, and it should be recognized that vigorous intensity activities may provide an even greater benefit. Aerobic-based activities that stress the cardiovascular and respiratory systems have the greatest health benefit, other than for bone health, in which case high-impact weight bearing activities are required.

## Competing interests

Production of this paper has been made possible through a financial contribution from the Public Health Agency of Canada. The views expressed herein do not necessarily represent the views of the Public Health Agency of Canada. I Janssen has received honoraria, speaker fees, and consulting fees from several non-profit organizations, including the Public Health Agency of Canada, that have an interest in physical activity and health.

## Authors' contributions

IJ designed the methods, assisted with the completion of the systematic review, and drafted the manuscript. AB lead most of the components of the systematic review and helped drafts some of the methodology sections of the paper.

All authors have read and approved the final manuscript.

## Supplementary Material

Additional file 1 **Table 1**. Association between physical activity and health and behavioural outcomes in children and youth. Click here for file

Additional file 2 **Table 2**. Criteria for assigning a level of evidence to recommendations. Click here for file

Additional file 3 **Table 3**. Criteria for assigning a grade to recommendations. Click here for file

Additional file 4 **Table 4**. Experimental studies examining the influence of exercise on changes in traditional blood lipids and lipoproteins in school-aged children and youth. Click here for file

Additional file 5 **Table 5**. Observational studies examining the relation between physical activity and fitness with hypertension in school-aged children and youth. Click here for file

Additional file 6 **Table 6**. Experimental studies examining the influence of exercise on changes in blood pressure in school-aged children and youth. Click here for file

Additional file 7 **Table 7**. Observational studies examining the relation between physical activity and fitness with the metabolic syndrome in school-aged children and youth. Click here for file

Additional file 8 **Table 8**. Experimental studies examining the influence of exercise on changes in markers of the metabolic syndrome (insulin resistance) in school-aged children and youth. Click here for file

Additional file 9 **Table 9**. Observational studies examining the relation between physical activity and fitness with obesity in school-aged children and youth. Click here for file

Additional file 10 **Table 10**. Experimental studies examining the influence of exercise on changes in obesity measures in school-aged children and youth. Click here for file

Additional file 11 **Table 11**. Experimental studies examining the influence of exercise on changes in bone mineral density in school-aged children and youth. Click here for file

Additional file 12 **Table 12**. Observational studies examining the relation between physical activity and fitness with depression in school-aged children and youth. Click here for file

Additional file 13 **Table 13**. Experimental studies examining the influence of exercise on changes in measures of depression in school-aged children and youth. Click here for file

Additional file 14 **Table 14**. Observational studies examining the relation between physical activity and fitness with injury in school-aged children and youth. Click here for file
