# Supplementary material for: Systematic review of the health benefits of physical activity and fitness in school-aged children and youth
Source: Int J Behav Nutr Phys Act. 2010 May 11;7:40. doi: 10.1186/1479-5868-7-40 (PMC2885312; doi:10.1186/1479-5868-7-40)
Supplement: Additional file 1 — Table 1. Association between physical activity and health and behavioural outcomes in children and youth. [file 1479-5868-7-40-S1.DOC]

**Table 1:** **Association between physical activity and health and behavioural outcomes in children and youth. Taken from I. Janssen [3].**

| **Health/Behavioural Outcome** | **Sample^a^** | **Effect^b^** | **Amount of Physical Activity Required for Health Benefit** |
| --- | --- | --- | --- |
| Adiposity | overweight or obese | + | F: 3-5 d/wk  I: moderate to vigorous  D: 30-40 min/d  T: variety of aerobic activities |
|  | normal weight | 0 | unclear ( probably similar to overweight) |
| Cardiovascular Health |  |  |  |
| Metabolic syndrome | overweight or obese | + | Unclear |
| Lipids/lipoproteins |  |  |  |
| Total cholesterol |  | 0 | Unclear |
| LDL-cholesterol |  | 0 | Unclear |
| HDL-cholesterol |  | + | unclear (probably similar to adiposity) |
| Triglycerides |  | + | unclear (probably similar to adiposity) |
| Blood pressure | normotensive | 0 | Unclear |
|  | Hypertensive | + | F: 12-32 wk, 3 d/wk  I: intensity to improve aerobic fitness  D: 30 min/session  T: aerobic |
| Endothelial function |  | 0 | Unclear |
| Inflammation |  | 0 | Unclear |
| Heart rate variability |  | 0 | Unclear |
| Coagulation |  | 0 | Unclear |
| Mental Health |  |  |  |
| Anxiety symptoms |  | + | unclear (varies with mode of activity) |
| Depression symptoms |  | + | unclear (varies with mode of activity) |
| Self-concept |  |  |  |
| Global self-concept |  | + | Unclear |
| Physical self-concept |  | + | Unclear |
| Sport competence |  | + | Unclear |
| Social self-concept |  | weak + | Unclear |
| Academic self-concept |  | weak + | Unclear |
| Academic performance |  |  |  |
| Grades, standardized tests |  | + | unclear (added physical education) |
| Indicators (eg, memory) |  | + | Unclear |
| Bone strength | prepubertal and pubertal | + | F: 2-3+ times/wk  I: moderate-high strain  D: 10-60 min  T: 10 min of impact, 45-60 min of general weight bearing |
|  | postpubertal | + weak | Unclear |
| Fitness |  |  |  |
| Aerobic fitness | >7 years | + | F: >3 d/wk  I: vigorous (80% VO_2_max)  D: 30-45 min  T: variety of activities |
| Strength and endurance | >6 years | + | F: 2-3 d/wk  I: Strength – 70-85% 1RM  Endurance – 30-60% 1RM  Sets – 2-5  D: 30-45 min  T: variety with adult supervision |

^a^ Unless otherwise indicated, all samples are from the general child and youth population

^b^ + = positive (beneficial) effect, 0 = null effect (insufficient evidence upon which to base a decision or no effect identified)

^c^ Amount of activity required to achieve the result. F = frequency, I = intensity, D = duration, T = type of activity.
