# Supplementary material for: Systematic review of the health benefits of physical activity and fitness in school-aged children and youth
Source: Int J Behav Nutr Phys Act. 2010 May 11;7:40. doi: 10.1186/1479-5868-7-40 (PMC2885312; doi:10.1186/1479-5868-7-40)
Supplement: Additional file 2 — Table 2. Criteria for assigning a level of evidence to recommendations. [file 1479-5868-7-40-S2.DOC]

**Table 2: Criteria for assigning a level of evidence to recommendations. Taken from Lau et al. [23]**

| **Level of Evidence** | **Criteria** |
| --- | --- |
| 1 | - Randomized controlled trials *withou*t important limitations. |
| 2 | - Randomized controlled trials *with* important limitations. - Observational studies with overwhelming evidence. |
| 3 | - Other observational studies. |
| 4 | - Inadequate data. - Anecdotal evidence or clinical experience. |
