# Supplementary material for: Systematic review of the health benefits of physical activity and fitness in school-aged children and youth
Source: Int J Behav Nutr Phys Act. 2010 May 11;7:40. doi: 10.1186/1479-5868-7-40 (PMC2885312; doi:10.1186/1479-5868-7-40)
Supplement: Additional file 3 — Table 3. Criteria for assigning a grade to recommendations. [file 1479-5868-7-40-S3.DOC]

**Table 3: Criteria for assigning a grade to recommendations. Taken from Lau et al. [23]**

| **Grade** | **Criteria** |
| --- | --- |
| A | Strong recommendation (action can apply to most individuals in most circumstances).   - Benefits clearly outweigh risks (or vice versa). - Evidence is level 1, 2, or 3. |
| B | Intermediate recommendation (action may vary depending on the person’s characteristics or other circumstances).   - Unclear whether benefits outweigh risk. - Evidence is level 1, 2, or 3. |
| C | Consensus (weak) recommendation (alternative actions may be equally reasonable).   - Unclear whether benefits outweigh risks. - Evidence is level 3 or 4. |
