# Supplementary material for: Systematic review of the health benefits of physical activity and fitness in school-aged children and youth
Source: Int J Behav Nutr Phys Act. 2010 May 11;7:40. doi: 10.1186/1479-5868-7-40 (PMC2885312; doi:10.1186/1479-5868-7-40)
Supplement: Additional file 4 — Table 4. Experimental studies examining the influence of exercise on changes in traditional blood lipids and lipoproteins in school-aged children and youth. [file 1479-5868-7-40-S4.DOC]

**Table 4:** **Experimental studies examining the influence of exercise on changes in traditional blood lipids and lipoproteins in school-aged children and youth.**

|  | |  | | Subject Characteristics | | | | | |  | Characteristics of Exercise Intervention | | | | | % Change in Outcomes** (* indicates significance) |  |  | | | | | | | |
| --- | --- | --- | --- | --- | --- | --- | --- | --- | --- | --- | --- | --- | --- | --- | --- | --- | --- | --- | --- | --- | --- | --- | --- | --- | --- |
| Reference | | Study Design | | N | | Sex | Ages | Nationality | Other |  | Type | Frequency  (days/wk) | Duration  (min) | Length (wk) | Intensity |  | Effect Size  (95% CI) |  | | | | | | | |
|  | |  | |  | |  |  |  |  |  |  |  |  |  |  |  |  |  | | | | | | | |
| *Aerobic Exercise Interventions* | | | | | | | | | | | |  |  |  |  |  |  |  |  |  |  |  |  |  |  |
| [29] | non-randomized | | 7 | | | males | mean | American | obese |  | aerobic | 3 | 20 | 15 | 60-70% | Total-C = -11.8% | -0.60 (-1.16, 0.10) |  | | | | | | | |
|  |  | |  | | |  | 13.3 |  |  |  |  |  |  |  | HR max | TG = +22.2% | 0.33 (-0.46, 0.81) |  | | | | | | | |
|  |  | |  | | |  |  |  |  |  |  |  |  |  |  | HDL-C = +16.9% | 0.73 (0.52, 0.82) |  | | | | | | | |
|  |  | |  | | |  |  |  |  |  |  |  |  |  |  | LDL-C = -25.2%* | -1.36 (-0.87, -1.72) |  | | | | | | | |
|  |  | |  | | |  |  |  |  |  |  |  |  |  |  |  |  |  | | | | | | | |
| [30] | RCT | | 36 | | | both | 9-12 | US Hispanic | high |  | aerobic | 3 | 60 | 6 | 75-80% | Total-C = -8.7% | -0.53 (-18.8, 20.4) |  | | | | | | | |
|  |  | |  | | |  |  |  | cholesterol |  |  |  |  |  | HR max | TG = -29.7%* | -1.10 (-26.6, 31.6) |  | | | | | | | |
|  |  | |  | | |  |  |  |  |  |  |  |  |  |  | HDL-C = -10.8% | 1.33 (0.03, 3.95) |  | | | | | | | |
|  |  | |  | | |  |  |  |  |  |  |  |  |  |  | LDL-C = +18.4% | 0.64 (-18.3, 18.3) |  | | | | | | | |
|  |  | |  | | |  |  |  |  |  |  |  |  |  |  |  |  |  | | | | | | | |
| [31] | RCT | | 28 | | | males | 10-14 | Greek |  |  | aerobic | 4 | 60 | 8 | 75% VO_2_max | HDL-C = +16.1%* | 0.78 (0.69, 0.92) |  | | | | | | | |
|  |  | |  | | |  |  |  |  |  |  |  |  |  |  |  |  |  | | | | | | | |
| [32] | RCT | | 79 | | | both | 7-11 | American | obese |  | aerobic | 5 | 40 | 20 | HR >150 bpm | Total-C = -5.8% | -0.49 (-0.65, -0.33) |  | | | | | | | |
|  |  | |  | | |  |  |  |  |  |  |  |  |  |  | TG = -22.6%* | -3.33 (-3.35, -3.31) |  | | | | | | | |
|  |  | |  | | |  |  |  |  |  |  |  |  |  |  | HDL-C = +4.4% | 0.26 (0.20, 0.32) |  | | | | | | | |
|  |  | |  | | |  |  |  |  |  |  |  |  |  |  | LDL-C = +3.2% | -0.29 (-0.45, -0.14) |  | | | | | | | |
|  |  | |  | | |  |  |  |  |  |  |  |  |  |  |  |  |  | | | | | | | |
| [36] | RCT | | 102 | | | both | 11-16 | German | obese |  | aerobic | 3 | 60 |  |  | TG = -26.2%* | -0.46 (-0.62, -0.07) |  | | | | | | | |
|  |  | |  | | |  |  |  |  |  |  |  |  |  |  | HDL-C = -0.9% | -0.04 (-0.12, 0.04) |  | | | | | | | |
|  |  | |  | | |  |  |  |  |  |  |  |  |  |  | LDL-C = -5.2%* | -0.21 (-0.43, 0.03) |  | | | | | | | |
|  |  | |  | | |  |  |  |  |  |  |  |  |  |  |  |  |  | | | | | | | |
|  |  | |  | | |  |  |  |  |  |  |  |  |  |  |  |  |  | | | | | | | |
| *Resistance Exercise and Circuit Training Interventions* | | | | | | | | | |  |  |  |  |  |  |  |  |  |  |  |  |  |  |  | |
| [33] | | RCT | | | 37 | both | 10-17 | Chinese |  |  | resistance | 3 | 60 | 6 | 70-85% 1 RM, | Total-C = -2.2% | -0.12 (-0.50, 0.22) |  | | | | | | | |
|  | |  | | |  |  |  |  |  |  |  |  |  |  | 3 sets of | TG = -7.1% | -0.14 (-0.44, 0.16) |  | | | | | | | |
|  | |  | | |  |  |  |  |  |  |  |  |  |  | 10 exercises | HDL-C = +8.3% | 0.40 (0.27, 0.49) |  | | | | | | | |
|  | |  | | |  |  |  |  |  |  |  |  |  |  |  | LDL-C = -3.6% | -0.14 (-0.44, 0.16) |  | | | | | | | |
|  | |  | | |  |  |  |  |  |  |  |  |  |  |  |  |  |  | | | | | | | |
| [34] | | non-randomized | | | 14 | both | mean |  | obese |  | circuit | 3 | 60 | 8 |  | Total-C = +5.7% | 0.23 (-0.11, 0.91) |  | | | | | | | |
|  | |  | | |  |  | 12.7 |  |  |  | training |  |  |  |  | TG = +14.5% | 0.35 (0.10, 0.63) |  | | | | | | | |
|  | |  | | |  |  |  |  |  |  |  |  |  |  |  | HDL-C = +3.7%% | 0.11 (-0.06, 0.34) |  | | | | | | | |
|  | |  | | |  |  |  |  |  |  |  |  |  |  |  | LDL-C = +4.1% | 0.12 (-0.22, 0.55) |  | | | | | | | |
|  | |  | | |  |  |  |  |  |  |  |  |  |  |  |  |  |  | | | | | | | |
|  | |  | | |  |  |  |  |  |  |  |  |  |  |  |  |  |  | | | | | | | |
| *Combined Aerobic and Resistance Exercise Interventions* | | | | | | | |  |  |  |  |  |  |  |  |  |  |  |  |  |  |  | | | |
| [35] | | RCT | | | 13 | female | mean | French | type 1 |  | aerobic + | 2 | 90 | 24 | 80-90% | Total-C = -5.1% | -0.33 (-0.74, 0.17) |  | | | | | | | |
|  | |  | | |  |  | 16.1 |  | diabetes |  | resistance |  |  |  | HR reserve | TG = -8.7% | -0.31 (-0.37, -0.12) |  | | | | | | | |
|  | |  | | |  |  |  |  |  |  |  |  |  |  |  | HDL-C = +1.3% | 0.06 (-0.14, 0.28) |  | | | | | | | |
|  | |  | | |  |  |  |  |  |  |  |  |  |  |  | LDL-C = -8.6% | -0.37 (-0.75 (0.07) |  | | | | | | | |
|  | |  | | |  |  |  |  |  |  |  |  |  |  |  |  |  |  | | | | | | | |

** the % change values represent within group % changes in mean values from pre- to post-treatment

RCT = randomized controlled trial; HR = heart rate; bpm = beats per minute; RM = repetition maximum; total-C = total cholesterol; HDL-C = high density lipoprotein cholesterol; LDL = low density lipoprotein cholesterol; TG = triglycerides.
