# Supplementary material for: Systematic review of the health benefits of physical activity and fitness in school-aged children and youth
Source: Int J Behav Nutr Phys Act. 2010 May 11;7:40. doi: 10.1186/1479-5868-7-40 (PMC2885312; doi:10.1186/1479-5868-7-40)
Supplement: Additional file 5 — Table 5. Observational studies examining the relation between physical activity and fitness with hypertension in school-aged children and youth. [file 1479-5868-7-40-S5.DOC]

**Table 5:**  **Observational studies examining the relation between physical activity and fitness with hypertension in school-aged children and youth.**

|  |  | **Subject Characteristics** | | | | **Physical Activity or Fitness Measurement** | **Odds or Hazard Ratio**  **(95% CI)**  **[least to most active]** |
| --- | --- | --- | --- | --- | --- | --- | --- |
| **Reference** | **Study Design** | **N** | **Sex** | Age (y) | **Ethnicity & Nationality** | **(Intensity)** |
|  |  |  |  |  |  |  |  |
| *Subjective Measures of Physical Activity* | | | | | |  |  |
| [37] | prospective | 2109 | both | 12-17 | Canadian | self-reported questionnaire | 0.92 (0.84, 1.0) |
|  | cohort |  |  |  |  | (all intensities) | 1.00 |
|  |  |  |  |  |  |  |  |
|  |  |  |  |  |  |  |  |
| *Cardiorespiratory Fitness* | | | |  |  |  |  |
| [28] | cross- | 3110 | both | 12-19 | mixed American | cardiorespiratory fitness | Females |
|  | sectional |  |  |  |  |  | unfit, 1.35 (0.68, 2.70) |
|  |  |  |  |  |  |  | fit, 1.00 |
|  |  |  |  |  |  |  | Males |
|  |  |  |  |  |  |  | unfit, 1.03 (0.30, 3.54) |
|  |  |  |  |  |  |  | fit, 1.00 |
|  |  |  |  |  |  |  |  |
| [38] | cross- | 13557 | both | mean 17.2 | Danish | cardiorespiratory fitness | Females |
|  | sectional |  |  |  |  |  | 1.5 (1.3, 1.8) |
|  |  |  |  |  |  |  | 1.1 (0.9, 1.4) |
|  |  |  |  |  |  |  | 1.0 (0.9, 1.3) |
|  |  |  |  |  |  |  | 1.1 (0.9, 1.3) |
|  |  |  |  |  |  |  | 1.00 |
|  |  |  |  |  |  |  | Males |
|  |  |  |  |  |  |  | 1.3 (1.1, 1.7) |
|  |  |  |  |  |  |  | 1.0 (0.8, 1.2) |
|  |  |  |  |  |  |  | 1.2 (0.9, 1.4) |
|  |  |  |  |  |  |  | 1.0 (0.8, 1.3) |
|  |  |  |  |  |  |  | 1.00 |
