# Supplementary material for: Systematic review of the health benefits of physical activity and fitness in school-aged children and youth
Source: Int J Behav Nutr Phys Act. 2010 May 11;7:40. doi: 10.1186/1479-5868-7-40 (PMC2885312; doi:10.1186/1479-5868-7-40)
Supplement: Additional file 6 — Table 6. Experimental studies examining the influence of exercise on changes in blood pressure in school-aged children and youth. [file 1479-5868-7-40-S6.DOC]

**Table 6: Experimental studies examining the influence of exercise on changes in blood pressure in school-aged children and youth.**

|  |  | | | Subject Characteristics | | | | | |  | Characteristics of Exercise Intervention | | | | | % Change in Outcomes ** (* indicates significance) | Effect Size  (95% CI) |  | | | | |
| --- | --- | --- | --- | --- | --- | --- | --- | --- | --- | --- | --- | --- | --- | --- | --- | --- | --- | --- | --- | --- | --- | --- |
| Reference | Study Design | | | N | Sex | | Age (y) | Nationality | Other |  | Type | Frequency  (days/wk) | Duration  (min) | Length (wk) | Intensity |  | | | | |
|  |  | | |  | |  |  |  |  |  |  |  |  |  |  |  |  |  | | | | |
| *Aerobic Exercise Interventions* | | | | | | | | |  |  |  |  |  |  |  |  |  |  |  |  |  |  |
| [29] | group | 7 | | | | male | mean | American | obese |  | aerobic | 3 | 20 | 15 | 60-70% | SBP = -5.7%* | -1.17 (-5.91, 2.68) |  | | | | |
|  | based |  | | | |  | 13.3 |  |  |  |  |  |  |  | HR max | DBP = -6.9% | -0.79 (-5.01, 4.91) |  | | | | |
|  |  |  | | | |  |  |  |  |  |  |  |  |  |  |  |  |  | | | | |
| [39] | non- | 25 | | | | both | 15-17 | American | high BP |  | aerobic | 5 | 30 | 24 | 70-80% | SBP = -5.8%* | -1.60 (-3.56, 0.36) |  | | | | |
|  | randomized |  | | | |  |  |  |  |  |  |  |  |  | VO_2_max | DBP = -6.3%* | -0.50 (-4.42, 3.42) |  | | | | |
|  |  |  | | | |  |  |  |  |  |  |  |  |  |  |  |  |  | | | | |
| [40] | RCT | 5 | | | | male | mean | American | high BP |  | aerobic | 3 | 50 | 25 | 60-75% | SBP = -9.1%* | -2.24 (-8.11, 2.05) |  | | | | |
|  |  |  | | | |  | 15.1 |  |  |  |  |  |  |  | VO_2_max | DBP = -3.8% | -0.43 (-6.30, 5.97) |  | | | | |
|  |  |  | | | |  |  |  |  |  |  |  |  |  |  |  |  |  | | | | |
| [41] | non- | 11 | | | | both | 8-12 | African- | high BP |  | aerobic | 3 | 30 | 12 | 67-80% | SBP = NS |  |  | | | | |
|  | randomized |  | | | |  |  | American |  |  |  |  |  |  | HR max | DBP = -10.7%* |  |  | | | | |
|  |  |  | | | |  |  |  |  |  |  |  |  |  |  |  |  |  | | | | |
| [42] | non- | 88 | | | | both | adol- | mixed | high BP |  | aerobic | 5 | 50 | 18 |  | SBP = -5.0%* | -0.97 (-2.83, 0.83) |  | | | | |
|  | randomized |  | | | |  | escent |  |  |  | classes |  |  |  |  | DBP = -1.9% | -0.16 (-2.14, 1.88) |  | | | | |
|  |  |  | | | |  |  |  |  |  |  |  |  |  |  |  |  |  | | | | |
|  |  |  | | | |  |  |  |  |  |  |  |  |  |  |  |  |  | | | | |
| *Non-Aerobic Exercise Interventions* | | | | | | | |  |  |  |  |  |  |  |  |  |  |  |  |  |  |  |
| [40] | RCT | | 6 | | | male | mean | American | high BP |  | resistance | 3 | 50 | 25 | 3 sets | SBP = * | -0.41 (-10.17, 5.43) |  | | | | |
|  |  | |  | | |  | 15.1 |  |  |  |  |  |  |  | 12-15 reps | DBP = NS | -0.41 (-10.17, 5.43) |  | | | | |
|  |  | |  | | |  |  |  |  |  |  |  |  |  | 14 exercises |  |  |  | | | | |
|  |  | |  | | |  |  |  |  |  |  |  |  |  |  |  |  |  | | | | |
| [33] | RCT | | 37 | | | both | 10-17 | Chinese |  |  | resistance | 3 | 60 | 6 | 70-85% 1 RM, | SBP = -5.4% | -0.67 (-5.12, 3.05) |  | | | | |
|  |  | |  | | |  |  |  |  |  |  |  |  |  | 3 sets | DBP = -5.8% | -0.47 (-3.90, 3.67) |  | | | | |
|  |  | |  | | |  |  |  |  |  |  |  |  |  | 10 exercises |  |  |  | | | | |
|  |  | |  | | |  |  |  |  |  |  |  |  |  |  |  |  |  | | | | |
| [34] | non- | | 14 | | | both | mean |  | obese |  | circuit training | 3 | 60 | 8 |  | SBP = -4.3% | -0.15 (-16.5, 15.2) |  | | | | |
|  | randomized | |  | | |  | 12.7 |  |  |  |  |  |  |  |  | DBP = -4.0%* | -0.89 (-16.3, 14.4) |  | | | | |
|  |  | |  | | |  |  |  |  |  |  |  |  |  |  |  |  |  | | | | |
| [43] | RCT | | 30 | | | female | 10-12 |  |  |  | pilates | 7 | 60 | 4 |  | SBP = -5.3%* | -0.76 (-3.50, 3.85) |  | | | | |
|  |  | |  | | |  |  |  |  |  |  |  |  |  |  | DBP = -6.1% | -0.58 (-3.96, 2.46) |  | | | | |
|  |  | |  | | |  |  |  |  |  |  |  |  |  |  |  |  |  | | | | |

** the % change values represent within group % changes in mean values from pre- to post-treatment

RCT = randomized controlled trial; HR = heart rate; BP = blood pressure; SBP = systolic blood pressure; DBP = diastolic blood pressure; NS = non-significant.
