# Supplementary material for: Systematic review of the health benefits of physical activity and fitness in school-aged children and youth
Source: Int J Behav Nutr Phys Act. 2010 May 11;7:40. doi: 10.1186/1479-5868-7-40 (PMC2885312; doi:10.1186/1479-5868-7-40)
Supplement: Additional file 7 — Table 7. Observational studies examining the relation between physical activity and fitness with the metabolic syndrome in school-aged children and youth. [file 1479-5868-7-40-S7.DOC]

**Table 7:** **Observational studies examining the relation between physical activity and fitness with the metabolic syndrome in school-aged children and youth.**

|  |  | **Subject Characteristics** | | | | **Physical Activity or Fitness Measurement** | **Odds or Hazard Ratio**  **(95% CI)**  **[least to most active]** |
| --- | --- | --- | --- | --- | --- | --- | --- |
| **Reference** | **Study Design** | **N** | **Sex** | **Age (y)** | **Ethnicity & Nationality** | **(Intensity)** |
|  |  |  |  |  |  |  |  |
| *Subjective Measures of Physical Activity* | | | | | |  |  |
|  |  |  |  |  |  |  |  |
| [45] | cross- | 352 | both | 14-19 | Mexican | self-reported questionnaire | 1.81 (0.72, 4.5) |
|  | sectional |  |  |  |  | (all intensities) | 1.00 |
|  |  |  |  |  |  |  |  |
| [48] | cross- | 1461 | both | 8-15 | mixed American | self-reported questionnaire | Females |
|  | sectional |  |  |  |  | (MVPA outside school) | 1.1 (0.6, 1.8) |
|  |  |  |  |  |  |  | 1.1 (0.6, 1.8) |
|  |  |  |  |  |  |  | 1.00 |
|  |  |  |  |  |  |  | Males |
|  |  |  |  |  |  |  | 1.6 (0.8, 2.7) |
|  |  |  |  |  |  |  | 1.3 (0.7, 2.4) |
|  |  |  |  |  |  |  | 1.00 |
|  |  |  |  |  |  |  |  |
| [49] | cross- | 4811 | both | 6-18 | Iranian | self-reported questionnaire | Females |
|  | sectional |  |  |  |  | (all intensities) | 1.6 (0.8, 1.7) |
|  |  |  |  |  |  |  | 1.03 (0.8, 1.7) |
|  |  |  |  |  |  |  | 1.00 |
|  |  |  |  |  |  |  | Males |
|  |  |  |  |  |  |  | 1.8 (1.1, 2.1) |
|  |  |  |  |  |  |  | 1.6 (1.08, 1.9) |
|  |  |  |  |  |  |  | 1.00 |
|  |  |  |  |  |  |  |  |
| *Objective Measures of Physical Activity* | | | | | | |  |
|  |  |  |  |  |  |  |  |
| [44] | cross- | 1732 | both | 9-15 | mixed European | accelerometry | 3.29 (1.96, 5.52) |
|  | sectional |  |  |  |  | (all intensities) | 3.13 (1.87, 5.25) |
|  |  |  |  |  |  |  | 2.51 (1.47, 4.26) |
|  |  |  |  |  |  |  | 2.03 (1.18, 3.5) |
|  |  |  |  |  |  |  | 1.00 |
|  |  |  |  |  |  |  |  |
| *Cardiorespiratory Fitness* | |  |  |  |  |  |  |
| [46] | cross- | 348 | both | 16-19 | Danish | cardiorespiratory fitness | 4.9 (1.7, 19.7) |
|  | sectional |  |  |  |  |  | 3.8 (0.9, 14.5) |
|  |  |  |  |  |  |  | 3.1 (1.0, 9.3) |
|  |  |  |  |  |  |  | 1.00 |
|  |  |  |  |  |  |  |  |
| [47] | prospective | 696 | both | 6-7 | Danish | cardiorespiratory fitness | 2.1 (1.0, 4.4) |
|  | cohort |  |  |  |  |  | 1.1 (0.5, 2.5) |
|  |  |  |  |  |  |  | 0.5 (0.2, 1.4) |
|  |  |  |  |  |  |  | 1.00 |
|  |  |  |  |  |  |  |  |
| [50] | cross- | 2844 | both | 9-15 | mixed European | cardiorespiratory fitness | Females |
|  | sectional |  |  |  |  |  | 10.4 (6.1, 17.8) |
|  |  |  |  |  |  |  | 4.3 (2.4, 7.5) |
|  |  |  |  |  |  |  | 2.1 (1.2-3.8) |
|  |  |  |  |  |  |  | 1.00 |
|  |  |  |  |  |  |  | Males |
|  |  |  |  |  |  |  | 15.8 (9.0, 27.6) |
|  |  |  |  |  |  |  | 5.3 (3.0, 9.5) |
|  |  |  |  |  |  |  | 2.9 (1.6, 5.3) |
|  |  |  |  |  |  |  | 1.00 |
|  |  |  |  |  |  |  |  |
| [28] | cross- | 3110 | both | 12-19 | mixed American | cardiorespiratory fitness | Females |
|  | sectional |  |  |  |  |  | unfit, 2.72 (0.85, 8.74) |
|  |  |  |  |  |  |  | fit, 1.00 |
|  |  |  |  |  |  |  | Males |
|  |  |  |  |  |  |  | unfit, 4.20 (2.14, 8.25) |
|  |  |  |  |  |  |  | fit, 1.00 |

MVPA = moderate-to-vigorous physical activity.
