# Supplementary material for: Systematic review of the health benefits of physical activity and fitness in school-aged children and youth
Source: Int J Behav Nutr Phys Act. 2010 May 11;7:40. doi: 10.1186/1479-5868-7-40 (PMC2885312; doi:10.1186/1479-5868-7-40)
Supplement: Additional file 8 — Table 8. Experimental studies examining the influence of exercise on changes in markers of the metabolic syndrome (insulin resistance) in school-aged children and youth. [file 1479-5868-7-40-S8.DOC]

**Table 8:**  **Experimental studies examining the influence of exercise on changes in markers of the metabolic syndrome (insulin resistance) in school-aged children and youth.**

|  |  | | Subject Characteristics | | | | | | | | |  | Characteristics of Exercise Intervention | | | | | % Change in Outcomes ** (* indicates significance) | Effect Size  (95 %CI) | | |  | | |  | | | | | | |
| --- | --- | --- | --- | --- | --- | --- | --- | --- | --- | --- | --- | --- | --- | --- | --- | --- | --- | --- | --- | --- | --- | --- | --- | --- | --- | --- | --- | --- | --- | --- | --- |
| Reference | Study Design | | N | | | Sex | | Age (y) | | Nationality | Other |  | Type | Frequency  (day/wk) | Duration  (min) | Length (wk) | Intensity |  | | |  | | | | | | |
|  |  | | |  | | |  | |  |  |  |  |  |  |  |  |  |  | | |  | | |  | | |  | | | | |
| *Aerobic Exercise Interventions* | | | | | | | | | |  |  |  |  |  |  |  |  |  | | |  | | |  | | |  |  |  |  |  |
| [32] | RCT | 79 | | | | both | | | 7-11 | American | obese |  | aerobic | 5 | 40 | 20 | HR | fasting IN = -9.3%* | | | -1.89 (-4.33, 0.56) | | |  | | |  | | | | |
|  |  |  | | | |  | | |  |  |  |  |  |  |  |  | >150 bpm |  | | |  | | |  | | |  | | | | |
|  |  |  | | | |  | | |  |  |  |  |  |  |  |  |  |  | | |  | | |  | | |  | | | | |
| [53] | non- | 19 | | | | female | | | mean | Greek | over- |  | aerobic | 3 | 40 | 12 | HR | fasting IN = +15.3% | | | 0.42 (-2.34, 2.43) | | |  | | |  | | | | |
|  | randomized |  | | | |  | | | 13.1 |  | weight |  |  |  |  |  | > 150 bpm | IN AUC = -23.3%* | | | -0.48 (1524, 2309) | | |  | | |  | | | | |
|  |  |  | | | |  | | |  |  |  |  |  |  |  |  |  | IN resistance = -19.0% | | | -0.05 (-0.39, 0.20) | | |  | | |  | | | | |
|  |  |  | | | |  | | |  |  |  |  |  |  |  |  |  |  | | |  | | |  | | |  | | | | |
| [36] | RCT | 102 | | | | both | | | 11-16 | German | obese |  | aerobic | 3 | 60 |  |  | fasting IN = -19.1%* | | | -0.54 (-2.11, 1.24) | | |  | | |  | | | | |
|  |  |  | | | |  | | |  |  |  |  |  |  |  |  |  | IN resistance = -20.8% | | | -0.52 (0.99, 0.07) | | |  | | |  | | | | |
|  |  |  | | | |  | | |  |  |  |  |  |  |  |  |  |  | | |  | | |  | | |  | | | | |
| [52] | randomized, | 50 | | | | both | | | mean | American | over- |  | fitness | 5 | 45 | 40 |  | fasting IN = -14.8%* | | | -0.26 (-5.54, 5.67) | | |  | | |  | | | | |
|  | non- |  | | | |  | | | 12.0 |  | weight |  | class |  |  |  |  |  | | |  | | |  | | |  | | | | |
|  | controlled |  | | | |  | | |  |  |  |  |  |  |  |  |  |  | | |  | | |  | | |  | | | | |
|  |  | | |  | |  | | |  |  |  |  |  |  |  |  |  |  | | |  | | |  | | |  | | | | |
| *Resistance Exercise and Circuit Training Interventions* | | | | | | | | | | |  |  |  |  |  |  |  |  | | |  | | |  | | |  |  |  |  |  |
| [54] | RCT | 22 | | | males | | | | mean | American | obese |  | resistance | 2 | 16 | 16 | progressive | fasting IN = -10.2% | | | -0.17 (-5.07, 3.94) | | |  | | |  | | | | |
|  |  |  | | |  | | | | 15.3 | Latino |  |  |  |  |  |  | & vigorous | IN resistance = -39.1%* | | | -0.91 (-1.49, -0.32) | | |  | | |  | | | | |
|  |  |  | | |  | | | |  |  |  |  |  |  |  |  |  |  | | |  | | |  | | |  | | | | |
| [55] | RCT | 22 | | | females | | | | 7-10 | American | obese |  | resistance | 3 | 20 | 22 |  | fasting IN = -4.1% | | | -0.08 (-31.2, 25.9) | | |  | | |  | | | | |
|  |  |  | | |  | | | |  |  |  |  |  |  |  |  |  | IN AUC = -8.2% | | | -0.21 (-63.0, 80.1) | | |  | | |  | | | | |
|  |  |  | | |  | | | |  |  |  |  |  |  |  |  |  |  | | |  | | |  | | |  | | | | |
| [33] | RCT | 37 | | | both | | | | 10-17 | Chinese |  |  | resistance | 3 | 60 | 6 | 70-85% | fasting IN = -2.0% | | | -0.29 (-0.46, -0.16) | | |  | | |  | | | | |
|  |  |  | | |  | | | |  |  |  |  |  |  |  |  | 1 RM |  | | |  | | |  | | |  | | | | |
|  |  |  | | |  | | | |  |  |  |  |  |  |  |  |  |  | | |  | | |  | | |  | | | | |
| [34] | non- | 14 | | | both | | | | mean |  | obese |  | circuit | 3 | 60 | 8 |  | fasting IN = -12.7% | | | -0.20 (-6.60, 8.32) | | |  | | |  | | | | |
|  | randomized |  | | |  | | | | 12.7 |  |  |  | training |  |  |  |  |  | |  | | |  | | |  | | | | | |

** the % change values represent within group % changes in mean values from pre- to post-treatment

RCT = randomized controlled trial; HR = heart rate; IN = insulin; AUC = area under the curve; NS = non-significant; PE = physical education.
