# Supplementary material for: Systematic review of the health benefits of physical activity and fitness in school-aged children and youth
Source: Int J Behav Nutr Phys Act. 2010 May 11;7:40. doi: 10.1186/1479-5868-7-40 (PMC2885312; doi:10.1186/1479-5868-7-40)
Supplement: Additional file 9 — Table 9. Observational studies examining the relation between physical activity and fitness with obesity in school-aged children and youth. [file 1479-5868-7-40-S9.DOC]

**Table 9:** **Observational studies examining the relation between physical activity and fitness with obesity in school-aged children and youth.**

|  |  | **Subject Characteristics** | | | | **Physical Activity or Fitness Measurement** | **Odds or Hazard Ratio**  **(95% CI)**  **[least to most active]** |
| --- | --- | --- | --- | --- | --- | --- | --- |
| **Reference** | **Study Design** | **N** | **Sex** | **Age (y)** | **Ethnicity & Nationality** | **(Intensity)** |
|  |  |  |  |  |  |  |  |
| *Subjective Measures of Physical Activity* | | | | | | | |
| [56] | cross- | 12759 | both | 11-19 | mixed American | self-reported questionnaire | Females |
|  | sectional |  |  |  |  | (MVPA) | 0.90 (0.85, 0.96) per weekly bout MVPA |
|  |  |  |  |  |  |  | Males |
|  |  |  |  |  |  |  | 0.86 (0.81, 0.91) per weekly bout MVPA |
|  |  |  |  |  |  |  |  |
| [57] | cross- | 15143 | both | 14-18 | mixed American | self-reported questionnaire | Females |
|  | sectional |  |  |  |  | (MVPA) | 1.24 (0.94, 1.63) |
|  |  |  |  |  |  |  | 1.25 (0.91, 1.7) |
|  |  |  |  |  |  |  | 1.00 |
|  |  |  |  |  |  |  | Males |
|  |  |  |  |  |  |  | 1.15 (0.97, 1.37) |
|  |  |  |  |  |  |  | 1.26 (1.01, 1.57) |
|  |  |  |  |  |  |  | 1.00 |
|  |  |  |  |  |  |  |  |
| [60] | prospective | 1083 | both |  | mixed American | self-reported questionnaire | Females |
|  | cohort |  |  |  |  | (active commuting to school) | 1.04 (0.52, 2.07) |
|  |  |  |  |  |  |  | 1.00 |
|  |  |  |  |  |  |  | Males |
|  |  |  |  |  |  |  | 1.10 (0.64, 1.92) |
|  |  |  |  |  |  |  | 1.00 |
|  |  |  |  |  |  |  |  |
| [61] | cross- | 1208 | both | 12-17 | Indian | self-reported questionnaire | 7.4% obese |
|  | sectional |  |  |  |  | (all intensities) | 5.6% obese |
|  |  |  |  |  |  |  | 0.0% obese |
|  |  |  |  |  |  |  |  |
| [62] | cross- | 461 | both | 9-16 | Mexican | self-reported questionnaire | 1.00 |
|  | sectional |  |  |  |  | (MVPA) | 0.96 (0.53, 1.74) |
|  |  |  |  |  |  |  | 0.92 (0.57, 1.49) |
|  |  |  |  |  |  |  | 0.7 (0.50, 0.98) |
|  |  |  |  |  |  |  |  |
| [63] | cross- | 7216 | both | 7-11 | mixed Canadian | parental report of organized | no, 1.00 |
|  | sectional |  |  |  |  | Sports (MVPA) | yes, 0.77 (0.65-0.91) |
|  |  |  |  |  |  |  |  |
| [64] | cross- | 5890 | both | 10-16 | mixed Canadian | self-reported questionnaire | Females |
|  | sectional |  |  |  |  | (MVPA) | 1.00 |
|  |  |  |  |  |  |  | 0.59 (0.34, 0.99) |
|  |  |  |  |  |  |  | 0.48 (0.28, 0.84) |
|  |  |  |  |  |  |  | 0.49 (0.25, 0.99) |
|  |  |  |  |  |  |  | Males |
|  |  |  |  |  |  |  | 1.00 |
|  |  |  |  |  |  |  | 0.53 (0.34, 0.84) |
|  |  |  |  |  |  |  | 0.28 (0.17, 0.46) |
|  |  |  |  |  |  |  | 0.29 (0.17, 0.49) |
|  |  |  |  |  |  |  |  |
| [65] | cross- | 4298 | both |  | mixed Canadian | self-reported questionnaire | 1.00 |
|  | sectional |  |  |  |  | (MVPA) | 0.93 (0.76, 1.15) |
|  |  |  |  |  |  |  | 0.89 (0.70, 1.08) |
|  |  |  |  |  |  |  | 0.74 (0.56, 0.97) |
|  |  |  |  |  |  |  |  |
| [66] | cross- | 1018 | both | 8-13 | mixed American | self-reported questionnaire | 0-59 min/d, 1.00 |
|  | sectional |  |  |  |  | (MVPA) | 60+ min/d, 0.59 (0.39, 0.89) |
|  |  |  |  |  |  |  |  |
| [68] | cross- | 6826 | both | 7-17 | Chinese | self-reported questionnaire | 0-44 min day, 1.00 |
|  | sectional |  |  |  |  | (MVPA) | 45+ min day, 0.80 (0.60, 1.0) |
|  |  |  |  |  |  |  |  |
| [69] | cross- | 445 | both | 11-16 | Tonga | self-reported questionnaire | 1.00 |
|  | sectional |  |  |  |  | (MVPA) | 0.55 (0.31, 0.99) |
|  |  |  |  |  |  |  | 0.61 (0.32, 1.12) |
|  |  |  |  |  |  |  |  |
| [70] | cross- | 1066 | both | 10-12 | mixed American | self-reported questionnaire | <2 hr/d, 24.7% overweight |
|  | sectional |  |  |  |  | (all intensities) | 2+ hr/d, 21.1% overweight |
|  |  |  |  |  |  |  |  |
| [71] | cross- | 1341 | both | 8-15 | Portugese | self-reported questionnaire | Female |
|  | sectional |  |  |  |  | (all intensities) | 1.1 (0.6, 1.9) |
|  |  |  |  |  |  |  | 0.8 (0.5, 1.5) |
|  |  |  |  |  |  |  | 1.00 |
|  |  |  |  |  |  |  | Male |
|  |  |  |  |  |  |  | 2.1 (1.0, 4.4) |
|  |  |  |  |  |  |  | 1.5 (0.8, 2.9) |
|  |  |  |  |  |  |  | 1.00 |
|  |  |  |  |  |  |  |  |
| [72] | case-control | 370 | both | 6-18 | Spanish | Interview (unclear) | 0.94 (0.92,0.96) per unit |
|  |  |  |  |  |  |  |  |
| [74] | cross- | 2389 | both | 10-16 | mixed American | self-reported questionnaire | Females |
|  | sectional |  |  |  |  | (all intensities) | Inactive, 1.00 |
|  |  |  |  |  |  |  | Average, 1.14 (0.79, 1.65) |
|  |  |  |  |  |  |  | Active (0.91 (0.61, 1.36) |
|  |  |  |  |  |  |  | Males |
|  |  |  |  |  |  |  | Inactive, 1.00 |
|  |  |  |  |  |  |  | Average, 0.65 (0.43, 0.98) |
|  |  |  |  |  |  |  | Active 0.54 (0.34, 0.79) |
|  |  |  |  |  |  |  |  |
| [77] | prospective | 1319 | both | 9-18 | Finnish | self-reported questionnaire | Females |
|  | cohort |  |  |  |  | (all intensities) | persistently inactive, 1.51 (0.32,5.99) |
|  |  |  |  |  |  |  | decreasingly active, 2.72 (1.04, 7.09) |
|  |  |  |  |  |  |  | increasingly active, 0.80 (0.29, 2.19) |
|  |  |  |  |  |  |  | persistently active, 1.00 |
|  |  |  |  |  |  |  | Males |
|  |  |  |  |  |  |  | persistently inactive, 0.87 (0.27, 2.85) |
|  |  |  |  |  |  |  | decreasingly active, 1.04 (0.41, 2.63) |
|  |  |  |  |  |  |  | increasingly active, 0.79 (0.32, 1.989) |
|  |  |  |  |  |  |  | Persistently active, 1.00 |
|  |  |  |  |  |  |  |  |
| [78] | case-control | 528 | both | 15-19 | Brazilian | self-reported questionnaire | inactive out of school, 1.00 |
|  |  |  |  |  |  | (unclear) | active out of school, 0.75 (0.44, 1.27) |
|  |  |  |  |  |  |  |  |
| [79] | cross- | 2108 | both | 9-12 | mixed Canadian | self-reported questionnaire | Females |
|  | sectional |  |  |  |  | (all intensities) | quintile 1, 13.9% obese |
|  |  |  |  |  |  |  | quintile 2, 13.0% obese |
|  |  |  |  |  |  |  | quintile 3, 13.4% obese |
|  |  |  |  |  |  |  | quintile 4, 12.8% obese |
|  |  |  |  |  |  |  | quintile 5, 13.3% obese |
|  |  |  |  |  |  |  | Males |
|  |  |  |  |  |  |  | quintile 1, 18.9% obese |
|  |  |  |  |  |  |  | quintile 2, 14.3% obese |
|  |  |  |  |  |  |  | quintile 3, 15.3% obese |
|  |  |  |  |  |  |  | quintile 4, 14.3% obese |
|  |  |  |  |  |  |  | quintile 5, 13.6% obese |
|  |  |  |  |  |  |  |  |
| [80] | cross- | 1890 | both | 12-16 | mixed American | self-reported questionnaire | * OR for healthy weight * |
|  | sectional |  |  |  |  | (MVPA) | 0 exercise programs, 1.00 |
|  |  |  |  |  |  |  | 1 program, 1.32 (0.86, 2.04) |
|  |  |  |  |  |  |  | 2 programs, 2.31 (1.47, 3.65) |
|  |  |  |  |  |  |  | 3+ program, 2.82 (1.76, 4.52) |
|  |  |  |  |  |  |  |  |
| [81] | cross- | 12538 | both | 9-14 | 12 European | self-reported questionnaire | Females |
|  | sectional |  |  |  | countries | (MVPA) | < 3 hr/wk, 1.17 (0.94, 1.46) |
|  |  |  |  |  |  |  | 3+ hr/wk, 1.00 |
|  |  |  |  |  |  |  | Males |
|  |  |  |  |  |  |  | < 3 hr/wk, 1.57 (1.29, 1.90) |
|  |  |  |  |  |  |  | 3+ hr/wk, 1.00 |
|  |  |  |  |  |  |  |  |
| [84] | cross- | 598 | both | 6-16 | Indian | self-reported questionnaire | tertile 1, 1.7 (0.8, 3.8) |
|  | sectional |  |  |  |  | (MVPA) | tertile 2, 0.8 (0.8, 3.8) |
|  |  |  |  |  |  |  | tertile 3, 1.00 |
|  |  |  |  |  |  |  |  |
| [85] | cross- | 240 | both | 8-10 | mixed American | self-reported questionnaire | low, 0.97 (0.45, 2.0) |
|  | sectional |  |  |  |  | (all intensities) | medium, 1.11 (0.58, 2.1) |
|  |  |  |  |  |  |  | high, 1.00 |
|  |  |  |  |  |  |  |  |
| [86] | cross- | 11265 | both | grade 8 & 10 | Latino American | self-reported questionnaire | 0.95 (0.91, 0.98) |
|  | sectional |  |  |  |  | (MVPA) | per day active |
|  |  |  |  |  |  |  |  |
| *Objective Measures of Physical Activity* | | | | | | | |
|  |  |  |  |  |  |  |  |
| [83] | cross- | 4661 | female | grade 6 & 8 | mixed American | accelerometry | Cross-sectional, Grade 6 |
|  | sectional & |  |  |  |  | (MVPA) | 15th %ile, 3.04 (1.79, 5.17) |
|  | longitudinal | 1968 |  |  |  |  | 85^th^ %ile, 1.00 |
|  |  |  |  |  |  |  | Cross-sectional, Grade 8 |
|  |  |  |  |  |  |  | 15th %ile, 2.35 (1.62, 3.39) |
|  |  |  |  |  |  |  | 85th %ile, 1.00 |
|  |  |  |  |  |  |  | Longitudinal, Gr. 6 and 8 |
|  |  |  |  |  |  |  | 15th %ile, 0.74 (0.43, 1.27) |
|  |  |  |  |  |  |  | 85th %ile, 1.00 |
|  |  |  |  |  |  |  |  |
| [73] | cross- | 608 | both | mean 9.6 | American | pedometer | Females, %overweight |
|  | sectional |  |  |  |  | (all intensities) | <10,000 steps, 40.9% |
|  |  |  |  |  |  |  | 10-12,000 steps, 22.7% |
|  |  |  |  |  |  |  | 12-14,000 steps, 26.0% |
|  |  |  |  |  |  |  | >14,000 steps, 10.8% |
|  |  |  |  |  |  |  | Males, % overweight |
|  |  |  |  |  |  |  | <10,000 steps, 40.4% |
|  |  |  |  |  |  |  | 10-12,000 steps, 29.2% |
|  |  |  |  |  |  |  | 12-14,000 steps, 23.1% |
|  |  |  |  |  |  |  | >14,000 steps, 1.38% |
|  |  |  |  |  |  |  |  |
| [59] | cross- | 248 | both | 8-11 | Swedish | accelerometry | 4.0 (1.2, 13.5) |
|  | sectional |  |  |  |  | (all intensities) | 3.7 (1.1, 12.4) |
|  |  |  |  |  |  |  | 0.7 (0.1, 3.2) |
|  |  |  |  |  |  |  | 1.00 |
|  |  |  |  |  |  |  |  |
| [67] | cross- | 5500 | both | 7-17 | UK | accelerometry | Females |
|  | sectional |  |  |  |  | (MVPA) | 1.00 |
|  |  |  |  |  |  |  | 0.36 (0.17, 0.74) |
|  |  |  |  |  |  |  | Males |
|  |  |  |  |  |  |  | 1.00 |
|  |  |  |  |  |  |  | 0.03 (0.01, 0.12) |
|  |  |  |  |  |  |  |  |
| *Fitness* | | | |  |  |  |  |
|  |  |  |  |  |  |  |  |
| [82] | cross- | 242 | both | 10-19 | Native Canadian | cardiorespiratory fitness | quartile 1, 1.00 |
|  | sectional |  |  |  |  |  | quartile 2, 0.52 (0.23, 1.16) |
|  |  |  |  |  |  |  | quartile 3, 0.24 (0.09, 0.66) |
|  |  |  |  |  |  |  | quartile 4, 0.13 (0.03, 0.48) |
|  |  |  |  |  |  |  |  |
| [75] | cross- | 602 | both | 11-14 | Australian | cardiorespiratory fitness | Females |
|  | sectional |  |  |  |  |  | 0.95 (0.91, 0.99) per lap |
|  |  |  |  |  |  |  | Males |
|  |  |  |  |  |  |  | 0.64 (0.47, 0.86) per lap |
|  |  |  |  |  |  |  |  |
| [76] | prospective | 918 | both | 6-12 | Greek | cardiorespiratory fitness | Unfit, 3.5 (1.62, 7.5) |
|  | cohort |  |  |  |  |  | Fit, 1.00 |
|  |  |  |  |  |  |  |  |
| [58] | cross- | 2211 | both | 5-14 | mixed American | fitness (aerobic + | Females |
|  | sectional |  |  |  |  | musculoskeletal) | unfit, 3.31 (1.95, 5.62) |
|  |  |  |  |  |  |  | fit, 1.00 |
|  |  |  |  |  |  |  | Males |
|  |  |  |  |  |  |  | unfit 1.79 (1.09, 3.00) |
|  |  |  |  |  |  |  | 1.00 |
|  |  |  |  |  |  |  |  |

MVPA = moderate-to-vigorous intensity physical activity
