# Supplementary material for: Systematic review of the health benefits of physical activity and fitness in school-aged children and youth
Source: Int J Behav Nutr Phys Act. 2010 May 11;7:40. doi: 10.1186/1479-5868-7-40 (PMC2885312; doi:10.1186/1479-5868-7-40)
Supplement: Additional file 10 — Table 10. Experimental studies examining the influence of exercise on changes in obesity measures in school-aged children and youth. [file 1479-5868-7-40-S10.DOC]

**Table 10:** **Experimental studies examining the influence of exercise on changes in obesity measures in school-aged children and youth.**

|  |  | Subject Characteristics | | | | |  | Characteristics of Exercise Intervention | | | | | % Change in Outcomes**  (* indicates significance) | Effect Size  (95% CI) |  | | | | |
| --- | --- | --- | --- | --- | --- | --- | --- | --- | --- | --- | --- | --- | --- | --- | --- | --- | --- | --- | --- |
| Reference | Study Design | N | Sex | Age (y) | Nationality | Other |  | Type | Frequency  (days/wk) | Duration  (min) | Length (wk) | Intensity |  | | | | |
|  |  |  |  |  |  |  |  |  |  |  |  |  |  |  |  | | | | |
| *Aerobic Exercise Interventions* | | | |  |  |  |  |  |  |  |  |  |  |  |  |  |  |  | |
| [29] | non- | 7 | male | mean | American | obese |  | aerobic | 3 | 20 | 15 | 60-70% | weight = +1.8%* | 0.16 (-6.87, 6.68) |  | | | | |
|  | randomized |  |  | 13.3 |  |  |  |  |  |  |  | HR max | % fat = -2.6% | -0.17 (-4.84, 3.90) |  | | | | |
|  |  |  |  |  |  |  |  |  |  |  |  |  |  |  |  | | | | |
| [39] | non- | 25 | both | 15-17 | American | high BP |  | aerobic | 5 | 30 | 24 | 70-80% | weight = +0.1% | 0.04 (-1.06, 1.13) |  | | | | |
|  | randomized |  |  |  |  |  |  |  |  |  |  | VO_2_max | skinfolds = -3.8% | -0.42 (-4.34, 3.11) |  | | | | |
|  |  |  |  |  |  |  |  |  |  |  |  |  |  |  |  | | | | |
| [42] | non- | 88 | both | adole- | Mixed | high BP |  | aerobics class |  | 50 | 18 |  | BMI = +1.2% | 0.05 (-1.63, 1.77) |  | | | | |
|  | randomized |  |  | scent |  |  |  |  |  |  |  |  |  |  |  | | | | |
|  |  |  |  |  |  |  |  |  |  |  |  |  |  |  |  | | | | |
| [32] | RCT | 79 | both | 7-11 | American | obese |  | aerobic | 5 | 40 | 20 | HR | % fat = -4.9%* | -0.88 (-1.66, -0.10) |  | | | | |
|  |  |  |  |  |  |  |  |  |  |  |  | >150 bpm |  |  |  | | | | |
|  |  |  |  |  |  |  |  |  |  |  |  |  |  |  |  | | | | |
| [53] | non- | 19 | female | mean | Greek | over- |  | aerobic | 3 | 40 | 12 | HR | BMI = -0.4% | -0.03 (-1.22, 1.20) |  | | | | |
|  | randomized |  |  | 13.1 |  | weight |  |  |  |  |  | > 150 bpm | % fat = -1.7% | -0.14 (-1.77, 1.37) |  | | | | |
|  |  |  |  |  |  |  |  |  |  |  |  |  | waist = +1.4% | 0.14 (-2.25, 2.80) |  | | | | |
|  |  |  |  |  |  |  |  |  |  |  |  |  |  |  |  | | | | |
| [35] | RCT | 13 | female | mean | French | type 1 |  | aerobic + | 2 | 90 | 24 | 80-90% | % fat = significant |  |  | | | | |
|  |  |  |  | 16.1 |  | diabetes |  | resistance |  |  |  | HR reserve |  |  |  | | | | |
|  |  |  |  |  |  |  |  |  |  |  |  |  |  |  |  | | | | |
| [36] | RCT | 102 | both | 11-16 | German | obese |  | aerobic | 3 | 60 |  |  | BMI = -8.7%* | -0.48 (-1.54, 2.21) |  | | | | |
|  |  |  |  |  |  |  |  |  |  |  |  |  | % fat = -2.8% | -0.12 (-3.48, 2.27) |  | | | | |
|  |  |  |  |  |  |  |  |  |  |  |  |  |  |  |  | | | | |
| [95] | RCT | 19 | both | mean | American | over- |  | aerobic | 4 | 30-50 | 8 | 50-80% | BMI = 0.0% | 0.00 (-1.76, 1.37) |  | | | | |
|  |  |  |  | 10.8 |  | weight |  |  |  |  |  | VO_2_max | % fat = +1.1% | 0.24 (-1.13, 1.61) |  | | | | |
|  |  |  |  |  |  |  |  |  |  |  |  |  | trunk fat = +2.8% | 0.57 (-1.00, 2.00) |  | | | | |
|  |  |  |  |  |  |  |  |  |  |  |  |  |  |  |  | | | | |
| [96] | RCT | 80 | both | 13-16 | American | obese |  | 1. moderate | 5 |  | 40 | 55-60% | - exercise reduced |  |  | | | | |
|  |  |  |  |  |  |  |  |  |  |  |  | V0_2_max | total & visceral fat |  |  | | | | |
|  |  |  |  |  |  |  |  | 2. vigorous | 5 |  | 40 | 75-80% | - no between group differences |  |  | | | | |
|  |  |  |  |  |  |  |  |  |  |  |  | VO_2_max |  |  |  | | | | |
|  |  |  |  |  |  |  |  |  |  |  |  |  |  |  |  | | | | |
|  |  |  |  |  |  |  |  |  |  |  |  |  |  |  |  | | | | |
| [97] | RCT | 74 | both | 7-11 | American | obese |  | aerobic | 5 | 40 | 18 | 70-75% | % fat = -3.1%* |  |  | | | | |
|  |  |  |  |  |  |  |  |  |  |  |  | HR max | % fat = -8.7%* |  |  | | | | |
|  |  |  |  |  |  |  |  |  |  |  |  |  | visceral fat = +0.5%* |  |  | | | | |
|  |  |  |  |  |  |  |  |  |  |  |  |  |  |  |  | | | | |
| [52] | randomized | 50 | both | mean | American | over- |  | fitness class | 5 | 45 | 40 |  | BMI = +3.1% | 0.13 (-3.65, 2.39) |  | | | | |
|  | (non- |  |  | 12.0 |  | weight |  |  |  |  |  |  | % fat = -10.7%* | -0.70 (3.12, 1.07) |  | | | | |
|  |  |  |  |  |  |  |  |  |  |  |  |  |  |  |  | | | | |
| *Resistance Exercise Training* | | | | | |  |  |  |  |  |  |  |  |  |  |  |  |  |  |
|  |  |  |  |  |  |  |  |  |  |  |  |  |  |  |  | | | | |
| [55] | RCT | 22 | females | 7-10 | American | obese |  | resistance | 3 | 20 | 22 |  | % fat = +0.8% | 0.05 (-3.44, 3.95) |  | | | | |
|  |  |  |  |  |  |  |  |  |  |  |  |  | trunk fat = +6.1% | 0.22 (-3.86, 4.24) |  | | | | |
|  |  |  |  |  |  |  |  |  |  |  |  |  | visceral fat = +2.7% | 0.06 (-13.6, 14.9) |  | | | | |
|  |  |  |  |  |  |  |  |  |  |  |  |  |  |  |  | | | | |
| [54] | RCT | 22 | males | mean | American | obese |  | resistance | 2 | 16 | 16 | progressive | weight = +2.1%* | 0.10 (-10.9, 11.1) |  | | | | |
|  |  |  |  | 15.3 | Latino |  |  |  |  |  |  | & vigorous | BMI = +0.9% | 0.06 (-3.08, 3.19) |  | | | | |
|  |  |  |  |  |  |  |  |  |  |  |  |  | % fat = -7.1%* | -0.34 (-4.45, 4.37) |  | | | | |
|  |  |  |  |  |  |  |  |  |  |  |  |  |  |  |  | | | | |
| [91] | RCT | 67 | female | 14-17 | American |  |  | resistance | 3 | 30-45 | 60 | 2-3 sets, | weight = +0.4% | -0.09 (-6.16, 7.54) |  | | | | |
|  |  |  |  |  |  |  |  |  |  |  |  | 9-10 reps | % fat = -8.9%* | -0.39 (-7.25, 6.27) |  | | | | |
|  |  |  |  |  |  |  |  |  |  |  |  | 15 exercises, |  |  |  | | | | |
|  |  |  |  |  |  |  |  |  |  |  |  |  |  |  |  | | | | |
| [88] | RCT | 32 | female | 14-18 | Canadian |  |  | resistance | 3 |  | 26 | 4 sets | weight = +1.2% | 0.07 (-5.61, 5.56) |  | | | | |
|  |  |  |  |  |  |  |  |  |  |  |  | 10-12 reps | % fat = -2.6% | -0.19 (-2.74, 2.16) |  | | | | |
|  |  |  |  |  |  |  |  |  |  |  |  | 13 exercises |  |  |  | | | | |
| *Other (Miscellaneous) Exercise Interventions* | | | |  |  |  |  |  |  |  |  |  |  |  |  |  |  |  | |
|  |  |  |  |  |  |  |  |  |  |  |  |  |  |  |  | | | | |
| [98] | non- | 425 | both | 6-10 | French |  |  | various | 2 | 60 | 26 | 70% | Females |  |  | | | | |
|  | randomized |  |  |  |  |  |  | exercises |  |  |  | HR max | BMI = significant |  |  | | | | |
|  |  |  |  |  |  |  |  |  |  |  |  |  | waist = significant |  |  | | | | |
|  |  |  |  |  |  |  |  |  |  |  |  |  | Males |  |  | | | | |
|  |  |  |  |  |  |  |  |  |  |  |  |  | BMI = significant |  |  | | | | |
|  |  |  |  |  |  |  |  |  |  |  |  |  | waist = NS |  |  | | | | |
|  |  |  |  |  |  |  |  |  |  |  |  |  |  |  |  | | | | |
| [43] | RCT | 30 | females | 10-12 |  |  |  | pilates | 7 | 60 | 4 |  | weight = -1.0% | -0.03 (-8.82, 9.18) |  | | | | |
|  |  |  |  |  |  |  |  |  |  |  |  |  | BMI = -2.3% | -0.09 (-2.83, 2.56) |  | | | | |
|  |  |  |  |  |  |  |  |  |  |  |  |  | Waist = -1.5% | -0.07 (-7.62, 7.67) |  | | | | |
|  |  |  |  |  |  |  |  |  |  |  |  |  |  |  |  | | | | |
| [93] | RCT | 90 | females | 6-8 | Swedish |  |  | PE classes | 5 | 40 | 104 |  | weight = +28.4% | 1.14 (-2.93, 3.88) |  | | | | |
|  |  |  |  |  |  |  |  |  |  |  |  |  | fat mass = +69.1% | 0.76 (-0.92, 1.88) |  | | | | |
|  |  |  |  |  |  |  |  |  |  |  |  |  |  |  |  | | | | |
| [42] | non- | 88 | both | adole- | mixed | high BP |  | PE classes |  |  |  |  | BMI = 0% | 0.00 (-1.51, 1.48) |  | | | | |
|  | randomized |  |  | scent |  |  |  |  |  |  |  |  |  |  |  | | | | |
|  |  |  |  |  |  |  |  |  |  |  |  |  |  |  |  | | | | |
| [87] | RCT | 21 | male | mean | Canadian | obese |  | lacrosse | 4 | 45 | 25 | sport | weight = +2.2% |  |  | | | | |
|  |  |  |  | 15.3 |  |  |  |  |  |  |  | participation | % fat = -7.0% |  |  | | | | |
|  |  |  |  |  |  |  |  |  |  |  |  |  | waist = +0.7% |  |  | | | | |
|  |  |  |  |  |  |  |  |  |  |  |  |  |  |  |  | | | | |
| [94] | RCT | 75 | females | 75 | Canadian |  |  | jumping | 3 | 12 | 80 |  | BMI = NS |  |  | | | | |
|  |  |  |  |  |  |  |  |  |  |  |  |  | fat mass = NS |  |  | | | | |
|  |  |  |  |  |  |  |  |  |  |  |  |  |  |  |  | | | | |
| [90] | RCT | 144 | female | 6-10 | Canadian |  |  | jumping & load | 3 | 30 | 32 |  | weight = +6.6% | 0.25 (-1.90, 2.21) |  | | | | |
|  |  |  |  |  |  |  |  | bearing |  |  |  |  | % fat = -0.3% | -0.04 (-1.80, 1.73) |  | | | | |
|  |  |  |  |  |  |  |  |  |  |  |  |  |  |  |  | | | | |
| [34] | non- | 14 | both | mean |  | obese |  | circuit training | 3 | 60 | 8 |  | BMI = -1.3% | -0.09 (-2.20, 2.19) |  | | | | |
|  | randomized |  |  | 12.7 |  |  |  |  |  |  |  |  | % fat = -1.2% | -0.10 (-3.16, 2.64) |  | | | | |
|  |  |  |  |  |  |  |  |  |  |  |  |  | waist = -2.3%* | -0.17 (-7.17, 7.01) |  | | | | |
|  |  |  |  |  |  |  |  |  |  |  |  |  |  |  |  | | | | |
| [92] | RCT | 64 | males | 8-12 | Canadian |  |  | circuit training | 3 | 12 | 80 | high | weight = NS |  |  | | | | |
|  |  |  |  |  |  |  |  |  |  |  |  | impact | BMI = NS |  |  | | | | |
|  |  |  |  |  |  |  |  |  |  |  |  |  | fat mass = NS |  |  | | | | |
|  |  |  |  |  |  |  |  |  |  |  |  |  |  |  |  | | | | |
| [89] | RCT | 71 | female | 9-10 | Australian |  |  | mixed | 3 | 30 | 30 | high | weight = -2.8% |  |  | | | | |
|  |  |  |  |  |  |  |  |  |  |  |  | impact | fat mass = -0.5% |  |  | | | | |
|  |  |  |  |  |  |  |  |  |  |  |  |  |  |  |  | | | | |
| [52] | randomized | 50 | both | mean | American | over- |  | 2. standard PE | 5 | 45 | 40 |  | BMI = 0.0% | 0.00 (-2.04, 1.63) |  | | | | |
|  | non- |  |  | 12.0 |  | weight |  |  |  |  |  |  | % fat = -5.2% | -0.37 (-2.74, 1.51) |  | | | | |
|  | controlled |  |  |  |  |  |  |  |  |  |  |  |  |  |  | | | | |
|  |  |  |  |  |  |  |  |  |  |  |  |  |  |  |  | | | | |

** the % change values represent within group % changes in mean values from pre- to post-treatment

RCT = randomized controlled trial; BP = blood pressure; PE = physical education; HR = heart rate; bpm = beats per minute; BMI = body mass index; NS = non-significant.
