# Supplementary material for: Systematic review of the health benefits of physical activity and fitness in school-aged children and youth
Source: Int J Behav Nutr Phys Act. 2010 May 11;7:40. doi: 10.1186/1479-5868-7-40 (PMC2885312; doi:10.1186/1479-5868-7-40)
Supplement: Additional file 11 — Table 11. Experimental studies examining the influence of exercise on changes in bone mineral density in school-aged children and youth. [file 1479-5868-7-40-S11.DOC]

**Table 11: Experimental studies examining the influence of exercise** on changes in bone mineral density in school-aged children and youth.

|  |  | | Subject Characteristics | | | | | | | |  | Characteristics of Exercise Intervention | | | | | | | |  | % Change in Outcomes ** (* indicates significance) | | Effect Size  (95% CI) |  | | | | | | | |
| --- | --- | --- | --- | --- | --- | --- | --- | --- | --- | --- | --- | --- | --- | --- | --- | --- | --- | --- | --- | --- | --- | --- | --- | --- | --- | --- | --- | --- | --- | --- | --- |
| Reference | Study Design | | N | | Sex | | Age (y) | | Nationality | |  | Type | | Frequency  (days/wk) | Duration  (min) | | Length (wk) | Intensity | |  |  | | | | | | | |
|  | |  | |  | |  | |  | |  | | |  | | |  | | |  | |  |  | | |  | |  | |  | |  |
| [100] | non- | | 99 | | both | | mean | | Finnish | |  | jumping | | 2 | 50 | | 39 |  | |  | lumbar spine BMD = +10.2%* | |  |  | | | | | | | |
|  | randomized | |  | |  | | 12.6 | |  | |  |  | |  |  | |  |  | |  | femoral neck BMD = +5.1% | |  |  | | | | | | | |
|  |  | |  | |  | |  | |  | |  |  | |  |  | |  |  | |  | trochanter BMD = +3.4% | |  |  | | | | | | | |
|  |  | |  | |  | |  | |  | |  |  | |  |  | |  |  | |  |  | |  |  | | | | | | | |
| [90] | RCT | | 144 | | female | | 6-10 | | Canadian | |  | jumping & | | 3 | 30 | | 32 |  | |  | total body BMD = +1.4% | | 0.23 (0.22, 0.24) |  | | | | | | | |
|  |  | |  | |  | |  | |  | |  | load | |  |  | |  |  | |  | trochanter BMD = +3.2%* | | 0.34 (0.33, 0.36) |  | | | | | | | |
|  |  | |  | |  | |  | |  | |  | bearing | |  |  | |  |  | |  | lumbar spine BMD = +1.9% | | 0.17 (0.16, 0.19) |  | | | | | | | |
|  |  | |  | |  | |  | |  | |  |  | |  |  | |  |  | |  | femoral neck BMD = +3.2* | | 0.28 (0.26, 0.30) |  | | | | | | | |
|  |  | |  | |  | |  | |  | |  |  | |  |  | | on |  | |  |  | |  |  | | | | | | | |
| [101, 102] | RCT | | 177 | | female | | mean | | Canadian | |  | jumping | | 3 | 10 | | 28 |  | |  | Prepubertal Youth | |  |  | | | | | | | |
|  |  | |  | |  | | 10.3 | |  | |  |  | |  |  | |  |  | |  | total body = +2.0 | |  |  | | | | | | | |
|  |  | |  | |  | |  | |  | |  |  | |  |  | |  |  | |  | lumbar spine = +4.6 | |  |  | | | | | | | |
|  |  | |  | |  | |  | |  | |  |  | |  |  | |  |  | |  | femur = +4.1 | |  |  | | | | | | | |
|  |  | |  | |  | |  | |  | |  |  | |  |  | |  |  | |  | Early Puberty Youth | |  |  | | | | | | | |
|  |  | |  | |  | |  | |  | |  |  | |  |  | |  |  | |  | total body = +3.8 | |  |  | | | | | | | |
|  |  | |  | |  | |  | |  | |  |  | |  |  | |  |  | |  | lumbar spine = +8.6* | |  |  | | | | | | | |
|  |  | |  | |  | |  | |  | |  |  | |  |  | |  |  | |  | femur = +6.5 | |  |  | | | | | | | |
|  |  | |  | |  | |  | |  | |  |  | |  |  | |  |  | |  |  | |  |  | | | | | | | |
| [103] | RCT | | 51 | | both | | 8-11 | | Canadian | |  | jumping | | 5 X 3 | 2 X 3 | | 24 |  | |  | hip BMD = +2.5% | |  |  | | | | | | | |
|  |  | |  | |  | |  | |  | |  |  | | sessions | sessions | |  |  | |  |  | |  |  | | | | | | | |
|  |  | |  | |  | |  | |  | |  |  | |  |  | |  |  | |  |  | |  |  | | | | | | | |
| [55] | RCT | | 75 | | female | | 75 | | Canadian | |  | jumping | | 3 | 12 | | 80 |  | |  | total body BMD = NS | |  |  | | | | | | | |
|  |  | |  | |  | |  | |  | |  |  | |  |  | |  |  | |  | lumbar spine BMD = NS | |  |  | | | | | | | |
|  |  | |  | |  | |  | |  | |  |  | |  |  | |  |  | |  |  | |  |  | | | | | | | |
| *Resistance Exercise* | | |  | |  | |  | |  | |  |  | |  |  | |  |  | |  |  | |  |  | |  | | | | | |
|  |  | |  | |  | |  | |  | |  |  | |  |  | |  |  | |  |  | |  |  | | | | | | | |
| [88] | RCT | | 32 | | female | | 14-18 | | Canadian | |  | resistance | | 3 |  | | 26 | 4 sets | |  | total body BMD = -0.2% | | -0.03 (-0.06, 0.00) |  | | | | | | | |
|  |  | |  | |  | |  | |  | |  |  | |  |  | |  | 9-10 reps | |  | lumbar spine BMD = +1.0% | | 0.08 (0.02, 0.14) |  | | | | | | | |
|  |  | |  | |  | |  | |  | |  |  | |  |  | |  | 13 exercises | |  |  | |  |  | | | | | | | |
|  |  | |  | |  | |  | |  | |  |  | |  |  | |  |  | |  |  | |  |  | | | | | | | |
| [91] | RCT | | 67 | | female | | 14-17 | | American | |  | resistance | | 3 | 30-45 | | 60 | 2-3 sets | |  | total body BMD = +2.8% | | 0.84 (0.81, 0.88) |  | | | | | | | |
|  |  | |  | |  | |  | |  | |  |  | |  |  | |  | 9-10 reps | |  | trochanter BMD = +2.1% | | 0.14 (0.02, 0.25) |  | | | | | | | |
|  |  | |  | |  | |  | |  | |  |  | |  |  | |  | 15 exercises | |  | lumbar spine BMD = +2.6% | | 0.39 (0.33, 0.46) |  | | | | | | | |
|  |  | |  | |  | |  | |  | |  |  | |  |  | |  |  | |  |  | |  |  | | | | | | | |
| *Other (Miscellaneous) Exercise Interventions* | | | | | | |  | |  | |  |  | |  |  | |  |  | |  |  | |  |  | |  | |  | |  | |
|  |  | |  | |  | |  | |  | |  |  | |  |  | |  |  | |  |  | |  |  | | | | | | | |
| [93] | RCT | | 90 | | female | | 6-8 | | Swedish | |  | PE classes | | 5 | 40 | | 104 |  | |  | total body BMD = +7.1% | | 1.33 (1.32, 1.34) |  | | | | | | | |
|  |  | |  | |  | |  | |  | |  |  | |  |  | |  |  | |  | femoral neck BMD = +11.1% | | 0.94 (0.92, 0.97) |  | | | | | | | |
|  |  | |  | |  | |  | |  | |  |  | |  |  | |  |  | |  |  | |  |  | | | | | | | |
| [89] | RCT | | 71 | | female | | 9-10 | | Australian | |  | mixed | | 3 | 30 | | 30 | high impact | |  | total body BMD = +3.5%* | |  |  | | | | | | | |
|  |  | |  | |  | |  | |  | |  |  | |  |  | |  |  | |  | lumbar spine BMD = +4.8%* | |  |  | | | | | | | |
|  |  | |  | |  | |  | |  | |  |  | |  |  | |  |  | |  | leg BMD = +6.8%* | |  |  | | | | | | | |
|  |  | |  | |  | |  | |  | |  |  | |  |  | |  |  | |  |  | |  |  | | | | | | | |
| [92] | RCT | | 64 | | male | | 8-12 | | Canadian | |  | circuits | | 3 | 12 | | 80 | high impact | |  | total body BMC = NS | |  |  | | | | | | | |
|  |  | |  | |  | |  | |  | |  |  | |  |  | |  |  | |  | femoral neck BMC = significant | |  |  | | | | | | | |
|  |  | |  | |  | |  | |  | |  |  | |  |  | |  |  | |  | lumbar spine BMC = NS | |  |  | | | | | | | |

** the % change values represent within group % changes in mean values from pre- to post-treatment

RCT = randomized controlled trial; BMD = bone mineral density; BMC = bone mineral content; NS = non-significant.
