# Supplementary material for: Systematic review of the health benefits of physical activity and fitness in school-aged children and youth
Source: Int J Behav Nutr Phys Act. 2010 May 11;7:40. doi: 10.1186/1479-5868-7-40 (PMC2885312; doi:10.1186/1479-5868-7-40)
Supplement: Additional file 12 — Table 12. Observational studies examining the relation between physical activity and fitness with depression in school-aged children and youth. [file 1479-5868-7-40-S12.DOC]

**Table 12:** **Observational studies examining the relation between physical activity and fitness with depression in school-aged children and youth.**

|  |  | **Subject Characteristics** | | | | **Physical Activity or Fitness Measurement** | **Odds or Hazard Ratio**  **(95% CI)**  **[least to most active]** |
| --- | --- | --- | --- | --- | --- | --- | --- |
| **Reference** | **Study Design** | **N** | **Sex** | **Age (y)** | **Ethnicity & Nationality** | **(Intensity)** |
|  |  |  |  |  |  |  |  |
| [104] | cross- | 1391 | both | 14-18 | mixed American | self-reported questionnaire | 1.0 |
|  | sectional |  |  |  |  | (vigorous) | 1.0 (0.76, 1.59) |
|  |  |  |  |  |  |  | 1.01 (0.75, 1.35) |
|  |  |  |  |  |  |  |  |
| [105] | cross- | 5453 | both | grades 7-11 | Chinese | self-reported questionnaire | 1.0 |
|  | sectional |  |  |  |  | (MVPA) | 0.55 (0.37, 0.83) |
|  |  |  |  |  |  |  | 0.84 (0.55, 1.28) |
|  |  |  |  |  |  |  |  |
| [106] | cross- | 509 | both | 15-19 | Finnish | interview | 1.48 (0.37, 2.12) |
|  | sectional |  |  |  |  | (not reported) | 0.88 (0.37, 2.12) |
|  |  |  |  |  |  |  | 1.0 |

MVPA = moderate-to-vigorous intensity physical activity
