# Supplementary material for: Systematic review of the health benefits of physical activity and fitness in school-aged children and youth
Source: Int J Behav Nutr Phys Act. 2010 May 11;7:40. doi: 10.1186/1479-5868-7-40 (PMC2885312; doi:10.1186/1479-5868-7-40)
Supplement: Additional file 13 — Table 13. Experimental studies examining the influence of exercise on changes in measures of depression in school-aged children and youth. [file 1479-5868-7-40-S13.DOC]

**Table 13:** **Experimental studies examining the influence of exercise on changes in measures of depression in school-aged children and youth.**

|  |  | Subject Characteristics | | | | |  | Characteristics of Exercise Intervention | | | | | % Change in Outcomes **  (* indicates significance) | Effect Size  (95% CI) |
| --- | --- | --- | --- | --- | --- | --- | --- | --- | --- | --- | --- | --- | --- | --- |
| Reference | Study Design | N | Sex | Age (y) | Nationality | Other |  | Type | Frequency  (days/wk) | Duration  (min) | Length (wk) | Intensity |
|  |  |  |  |  |  |  |  |  |  |  |  |  |  |  |
| [107] | RCT | 90 | both | 9-12 | American |  |  | aerobic | 3 | 45 | 12 | 55-80% | depression = -44.2%* | -0.58 (-1.40, 0.42) |
|  |  |  |  |  |  |  |  |  |  |  |  | HR max | mood disorder = -33.9%* | -0.43 (-3.47, 4.12) |
|  |  |  |  |  |  |  |  |  |  |  |  |  |  |  |
| [108] | RCT | 60 | both | 13-17 | American |  |  | 1. vigorous | 2 | 30 | 10 | 70-75% | 1. depression = -13.5%* | -0.27 (-2.44, 1.94) |
|  |  |  |  |  |  |  |  |  |  |  |  | HR max | stress = -13.3%* | -0.17 (-20.0, 16.4) |
|  |  |  |  |  |  |  |  | 2. moderate | 2 | 30 | 10 | 50-60% | 2. depression = +10.5% | 0.31 (-0.67, 2.57) |
|  |  |  |  |  |  |  |  |  |  |  |  | HR max | stress = -3.5% | -0.02 (-17.8, 19.4) |
|  |  |  |  |  |  |  |  | 3. flexibility | 2 | 30 | 10 |  | 3. depression = +49.1% | 0.77 (-1.25, 2.71) |
|  |  |  |  |  |  |  |  |  |  |  |  |  | stress = +14.6% | -0.01 (-26.1, 24.1) |
|  |  |  |  |  |  |  |  |  |  |  |  |  |  |  |
| [109] | RCT | 30 | both | 8-12 | Canadian | over- |  | behaviour |  |  | 8 |  | physical self worth = +19.0%* | 0.89 (-0.42, 2.42) |
|  |  |  |  |  |  | weight |  | modification |  |  |  |  | global self worth = +5.6% | 0.22 (-1.67, 2.24) |
|  |  |  |  |  |  |  |  | to increase activity |  |  |  |  |  |  |

** the % change values represent within group % changes in mean values from pre- to post-treatment

RCT = randomized controlled trial; HR = heart rate; NS = non-significant.
