# Supplementary material for: Systematic review of the health benefits of physical activity and fitness in school-aged children and youth
Source: Int J Behav Nutr Phys Act. 2010 May 11;7:40. doi: 10.1186/1479-5868-7-40 (PMC2885312; doi:10.1186/1479-5868-7-40)
Supplement: Additional file 14 — Table 14. Observational studies examining the relation between physical activity and fitness with injury in school-aged children and youth. [file 1479-5868-7-40-S14.DOC]

**Table 14:** **Observational studies examining the relation between physical activity and fitness with njury in school-aged children and youth.**

|  |  | **Subject Characteristics** | | | | **Physical Activity or Fitness Measurement** | **Odds or Hazard Ratio**  **(95% CI)**  **[least to most active]** |
| --- | --- | --- | --- | --- | --- | --- | --- |
| **Reference** | **Study Design** | **N** | **Sex** | **Age (y)** | **Ethnicity & Nationality** | **(Intensity)** |
|  |  |  |  |  |  |  |  |
| [114] | cross- | 8135 | both | 12-18 | Finish | self-reported questionnaire | 1.00 |
|  | sectional |  |  |  |  | (vigorous sports) | 1.1 (0.9-1.5) |
|  |  |  |  |  |  |  | 1.8 (1.4-2.3) |
|  |  |  |  |  |  |  | 3.3 (2.3-4.6) |
|  |  |  |  |  |  |  |  |
| [113] | cross- | 10222 | both | 12-15 | Taiwan | parental report | Females |
|  | sectional |  |  |  |  | (MVPA) | 1.00 |
|  |  |  |  |  |  |  | 1.4 (1.2-1.6) |
|  |  |  |  |  |  |  | 2.2 (1.8-2.6) |
|  |  |  |  |  |  |  | Males |
|  |  |  |  |  |  |  | 1.00 |
|  |  |  |  |  |  |  | 1.1 (0.9-1.4) |
|  |  |  |  |  |  |  | 1.7 (1.4-2.2) |
|  |  |  |  |  |  |  |  |
| [112] | cross- | 5559 | both | 10-17 | Canadian | self-reported questionnaire | Gr. 6-8 Activity in School |
|  | sectional |  |  |  |  | (MVPA) | 1.00 |
|  |  |  |  |  |  |  | 1.22 (0.89-1.68) |
|  |  |  |  |  |  |  | 1.35 (0.99-1.84) |
|  |  |  |  |  |  |  | Gr.9-10 Activity in School |
|  |  |  |  |  |  |  | 1.00 |
|  |  |  |  |  |  |  | 1.10 (0.70-1.75) |
|  |  |  |  |  |  |  | 1.46 (0.94-2.28) |
|  |  |  |  |  |  |  | Gr. 6-8 Activity out of School |
|  |  |  |  |  |  |  | 1.00 |
|  |  |  |  |  |  |  | 1.27 (1.04-1.55) |
|  |  |  |  |  |  |  | 1.97 (1.63-2.39) |
|  |  |  |  |  |  |  | Gr.9-10 Activity out of School |
|  |  |  |  |  |  |  | 1.00 |
|  |  |  |  |  |  |  | 1.65 1.23-2.24) |
|  |  |  |  |  |  |  | 2.98 (2.25-3.97) |
